# Development of an Influenza/COVID-19 Combination mRNA Vaccine Containing a Novel Multivalent Antigen Design That Enhances Immunogenicity of Influenza Virus B Hemagglutinins

**DOI:** 10.3390/vaccines13060628

**Published:** 2025-06-11

**Authors:** Elena Thornhill-Wadolowski, Dana L. Ruter, Feng Yan, Mayur Gajera, Evan Kurt, Labannya Samanta, Kimberlin Leigh, Jianbo Zhu, Zhijun Guo, Zihao Wang, Yuanqing Liu, Jaewoo Lee, Marcin Bugno

**Affiliations:** 1 Immorna Biotherapeutics, Inc., Morrisville, NC 27560, USA; elena.thornhill@immornabio.com (E.T.-W.); dana.ruter@immornabio.com (D.L.R.); evan.kurt@immornabio.com (E.K.); labannya.samanta@immornabio.com (L.S.); kimberlin.leigh@immornabio.com (K.L.); jaewoo.lee@immornabio.com (J.L.); 2 Immorna (Hangzhou) Biotechnology, Co., Ltd., Hangzhou 311215, China; jianbo.zhu@immorna.com (J.Z.); zhijun.guo@immorna.com (Z.G.); zihao.wang@immornabio.com (Z.W.); 3 Immorna (Shanghai) Biotechnology, Co., Ltd., Shanghai 201108, China; yuanqing.liu@immorna.com

**Keywords:** seasonal influenza mRNA vaccine, influenza/COVID-19 combination vaccine, multimeric antigens, hemagglutinin

## Abstract

Background/Objectives: Developing next-generation mRNA-based seasonal influenza vaccines remains challenging, primarily because of the relatively low immunogenicity of influenza B hemagglutinin (HA) antigens. We describe a systematic vaccine development strategy that combined vector and antigen design optimization. Methods: Novel untranslated region (UTR) sequences and a hybrid poly(A) tail were used to increase plasmid stability and mRNA expression. Fusion proteins containing HA antigens linked by T4 foldon domains were engineered to enhance the immune responses against influenza B HA antigens and to permit the expression of multiple HA ectodomains from a single mRNA species. The vaccine performance was verified in a traditional encapsulated lipid nanoparticle (LNP) formulation that requires long-term storage at temperatures below −15 °C as well as in a proprietary thermo-stable LNP formulation developed for the long-term storage of the mRNA vaccine at 2–8 °C. Results: In preclinical studies, our next-generation seasonal influenza vaccine tested alone or as a combination influenza/COVID-19 mRNA vaccine elicited hemagglutination inhibition (HAI) titers significantly higher than Fluzone HD, a commercial inactivated influenza vaccine, across all 2024/2025 seasonal influenza strains, including the B/Victoria lineage strain. At the same time, the combination mRNA vaccine demonstrated superior neutralizing antibody titers to 2023/2024 Spikevax, a commercial COVID-19 comparator mRNA vaccine. Conclusions: Our data demonstrate that the multimerization of antigens expressed as complex fusion proteins is a powerful antigen design approach that may be broadly applied toward mRNA vaccine development.

## 1. Introduction

Influenza viruses cause moderate to severe respiratory disease, resulting in an estimated 5000 to 50,000 deaths annually in the U.S. alone [1,2,3]. Due to the high disease burden, the development of next-generation influenza vaccines, including mRNA-based vaccines, is a public health priority and an active area of research. The main antigenic target for influenza is the homo-trimeric HA glycoprotein, which is expressed on the surface of the virion and fuses the viral and cell membranes to allow viral entry [4]. To broadly protect the general population, seasonal influenza vaccines include antigens from both type A and B influenza virus strains [5]. Seasonal influenza mRNA vaccines that are currently undergoing Phase 2 and Phase 3 clinical trials are composed of individual mRNAs, each encoding native-like membrane-bound HA protein antigens [6,7,8].

Poor type B influenza HA antigen responses have been of special concern in the development of mRNA-based influenza vaccines. Moderna, Pfizer, and GSK have all struggled with generating strong immunity against B type antigens. Moderna initially failed to meet non-inferiority criteria for both seroconversion rates and HAI titers for the influenza B-Victoria and B-Yamagata lineage strains in the Phase 3 trial for mRNA-1010 [9]. Pfizer–BioNTech also failed to meet non-inferiority criteria against B-Victoria in their Phase 3 trial for an influenza/COVID-19 combination vaccine [6]. GSK/CureVac’s interim Phase 2 data showed that the B-strain responses were lower for their mRNA vaccine compared with the commercial comparator’s [8]. Similarly, in Immorna’s first-generation quadrivalent mRNA vaccine JCXH-107, we needed to increase the relative content of B influenza strain HA encoding mRNAs to improve IgG and HAI titers against B/Victoria and B/Yamagata lineage strains [10].

We have recently developed a bivalent COVID-19 mRNA vaccine that encodes two SARS-CoV-2 antigens: the spike (S) protein receptor binding domain (RBD) of the ancestral strain and the Omicron BA.1 variant with additional L452R and F486V mutations found in Omicron BA.4/.5. Both RBD antigens are expressed from a single mRNA species as a secreted bivalent “dumbbell” fusion protein. A bacteriophage T4 foldon domain links the two RBD antigens within the “dumbbell” structure and facilitates their folding and trimerization [11]. The T4 foldon domain has been used to stabilize trimeric antigens in the recently licensed Pfizer and GSK RSV vaccines, including the pre-fusion form of the trimeric RSV F protein [12,13,14,15].

Conformational stabilization of trimeric antigens can result in more robust immune responses and generate more effective neutralizing antibodies [16,17]. Multiple methods, including the addition of a C-terminal T4 foldon domain, have been used to stabilize influenza HA trimers, especially when expressed as secreted recombinant antigens [16,18,19,20,21,22]. We decided to apply the “dumbbell” antigen design from our COVID-19 vaccine to develop next-generation mRNA influenza vaccines with two or three HA antigens encoded by a single mRNA template. This was expected to improve the immunogenicity of the conformationally stabilized antigens as well as to simplify the manufacturing of the multivalent vaccine.

**Figure 1:**
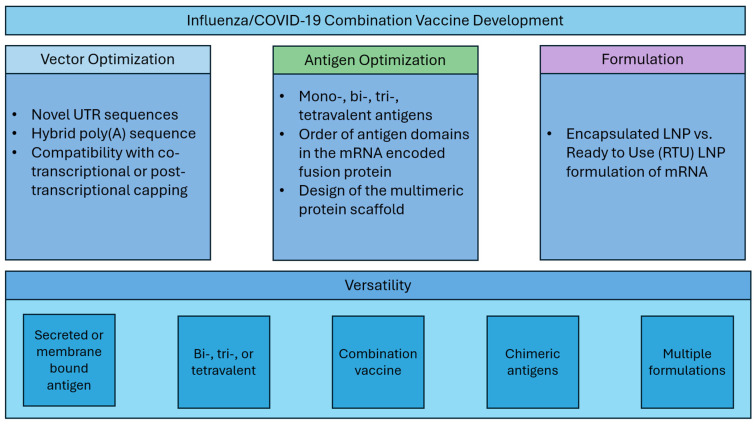
Vaccine development strategy.

Here, we describe systematic optimization of seasonal influenza mRNA vaccine candidates that encompasses (1) vector development with novel UTR and hybrid poly(A) sequences, (2) modular dumbbell HA antigen design with two or three HA antigens expressed from a single mRNA species, and (3) assessment of the mRNA vaccine compatibility with thermally stabilized mRNA formulations (Figure 1). Finally, we demonstrate that our next-generation seasonal influenza mRNA vaccine used in combination with the COVID-19 mRNA vaccine is highly immunogenic and outperforms commercial comparator influenza and COVID-19 vaccines in mouse preclinical studies.

## 2. Materials and Methods

### 2.1. Plasmids Construction

#### 2.1.1. Luciferase Reporter Plasmids

Synthetic DNA fragments, including the luciferase gene, 5′ or 3′ UTR sequences, and ultramers containing A_30_ and T_2_A_30_ blocks (IDT), were cloned into a pUC-GW-Kan plasmid (Azenta, Morrisville, NC, USA).

#### 2.1.2. Plasmids Coding for HA and S-Protein RBD Antigens

Amino acid sequences of influenza HA and COVID-19 XBB.1.5 S-protein RBD were downloaded from the Global Initiative on Sharing Avian Influenza Data (GSAID) EpiFlu and EpiCoV databases. The antigens derived from the seasonal influenza strains recommended by the World Health Organization (WHO) for cell-based vaccines in the northern hemisphere used in the study are listed in Table 1 [23,24,25,26].

The HA and XBB.1.5 RBD sequences were codon optimized for expression in human cells prior to cloning. Plasmid cloning and propagation were performed in *E. coli* Stbl3^TM^ or Stable^TM^ cells (Invitrogen, Waltham, MA, USA and New England Biolabs, Ipswitch, MA, USA) grown in kanamycin-LB media at 30 °C overnight. All plasmids were verified by Sanger sequencing. The sequences of individual antigens encoded by these plasmids are described in Supplemental Table S1.

### 2.2. Plasmid Stability

In order to compare A_120_ and A_30_(T_2_A_30_)_3_ tail stability, *E. coli* Stbl3^TM^ cells were transformed with luciferase reporter plasmids (pLuc) containing either homopolymeric poly(A) sequence (A_120_) or a hybrid poly(A) sequence including T_2_ spacers (A_30_(T_2_A_30_)_3_). After transformation, cells were plated on kanamycin selection plates and incubated at 37 °C overnight. Colonies were chosen at random and incubated in LB media at 37 °C overnight. Plasmids were purified from overnight cultures and analyzed by Sanger sequencing.

### 2.3. mRNA Production

Plasmids were linearized via BspQI and purified by phenol–chloroform extraction followed by sodium acetate precipitation and three 70% ethanol washes. The pellets were resuspended in nuclease-free water. mRNA was generated by in vitro transcription (IVT) using linear plasmid DNA template (50 µg/mL) and IVT reaction mixture containing 40 mM Tris-HCl, 24 mM MgCl_2_ (Invitrogen), ribonucleoside triphosphate mix (6 mM each NTP, with N1-methylpsuedouridine 5′-triphosphate used in place of UTP), 2 U/mL yeast inorganic pyrophosphatase, 1000 U/mL RNase inhibitor, and 5000 U/mL T7 RNA Polymerase (Hongene Biotech, Shanghai, China) at 30 °C for 3 h. The DNA template in the IVT reaction was removed by DNase (Hongene Biotech) treatment at 30 °C for 30 min. After DNase treatment, mRNA was precipitated with LiCl (Invitrogen). The pellets were washed in 70% ethanol, air-dried, and resuspended in nuclease-free water. Post-transcriptional capping reaction included the following components: 200 U/mL vaccinia capping system, capping buffer including 2 mM MgCl_2_, 0.75 mM GTP and 0.32 mM S-adenosylmethionine, 500 U/mL RNase inhibitor, and 1000 U/mL 2’-O-methyltransferase (all from Hongene Biotech). The capping reaction was carried out by incubating mRNA with vaccinia virus capping enzyme and 2-O-methyltransferase (Hongene Biotech) at 37 °C for 1.5 h, which added a 7-methylguanylate cap structure (Cap 1) to the 5′ end of mRNA. Newly capped mRNA was precipitated with LiCl (Invitrogen), and the pellets were washed in 70% ethanol, air-dried, and resuspended in nuclease-free water at a concentration of 1 μg/µL. The mRNA concentration was confirmed using a NanoDrop instrument. The correct size and mRNA purity were determined by RNA gel electrophoresis. mRNA was stored at −80 °C.

### 2.4. Formulation

#### 2.4.1. Ready to Use (RTU) Lipid Nano Particles (LNP)

The RTU LNP mRNA vaccines were formulated in a two-vial system composed of a lyophilized mRNA vial and a separate LNP dispersion vial. For the generation of combination vaccines comprising several mRNA species, the individual mRNA preparations were pre-mixed prior to lyophilization. The LNP dispersion was generated using a microfluidic process (Dolomite Microfluidics, Unchained Labs, Pleasanton, CA, USA). Four LNP components: 2-dimyristoyl-rac-glycero-3-methoxypolyethylene glycol-2000 (Avanti, Alabaster, AL, USA), cholesterol (Sigma-Aldrich, St. Louis, MO, USA), 1,2-distearoyl-sn-glycero-3-phosphocholine (Avanti, Alabaster, AL, USA), and cationic lipid (in-house code XH-07) were used at a molar ratio of 1.5%:40.5%:10%:48%. The LNP preparation was then concentrated by tangential flow filtration (TFF) (KrosFlo, Repligen, Waltham, MA), buffer-exchanged to the final formulation buffer (10mM citrate, 3.5% sucrose, pH 5.5), and characterized (particle size, polydispersity index, mRNA encapsulation rate; refer to Supplemental Materials and Methods). LNP preparations were stored at 4 °C.

Before use, the lyophilized mRNA and LNP dispersion vials were equilibrated to room temperature for 15 min. The LNP dispersion was gently shaken for 5–10 s, extracted by a syringe, and added to the lyophilized mRNA cake. After addition, the vial was inverted repeatedly for approximately 30 s to mix. The reconstituted vaccine suspension was injected intramuscularly.

All LNP batches used in this study met development specifications indicated as lower and upper limits shown in Supplemental Figures S2–S4.

#### 2.4.2. Encapsulated LNP

A microfluidic process was used to encapsulate mRNA within an LNP dispersion (Nanoassemblr Ignite, Cytiva, Marlborough, MA, USA). For the generation of combination vaccines comprising several mRNA species, the individual mRNA preparations were pre-mixed prior to encapsulation. mRNA and the four lipid components: 2-dimyristoyl-rac-glycero-3-methoxypolyethylene glycol-2000 (Avanti), cholesterol (Sigma-Aldrich), 1,2-distearoyl-sn-glycero-3-phosphocholine (Avanti), and cationic lipid (in-house code XH-07) were used at a molar ratio of 2%:48%:10%:40%. The generated LNP preparation was then dialyzed to the final formulation buffer (20 mM Tris, 5 mM NaCl, 7.5% sucrose, pH 7.2) using a Spectra/Por^®^ 3 Standard RC membrane (Repligen), concentrated using an Amicon^®^ 100 kDa molecular weight cut-off ultracentrifugal filter (Merck, Burlington, MA, USA), and characterized (particle size, polydispersity index, mRNA encapsulation rate). Encapsulated LNP mRNA vaccine vials were stored frozen at −80 °C prior to use.

Before use, the LNP dispersion was equilibrated to room temperature for 15 min and gently shaken for 5–10 s. The encapsulated LNP mRNA vaccine suspension was injected intramuscularly.

A comparison of the microfluidic mixing conditions, buffer exchange, and concentration parameters between the RTU and encapsulated LNP formulations can be seen in Supplemental Table S3A,B.

### 2.5. Cell Culture

BHK-21 and MDCK cells were purchased from ATCC (Manassas, VA, USA). 293T-ACE2 cells were purchased from Vazyme (Nanjing, China). Cells were cultured in Dulbecco’s modified Eagle’s medium (DMEM) supplemented with 10% fetal bovine serum (FBS) and 1% penicillin–streptomycin (Invitrogen). Cells were cultured in a humidified incubator with 5% CO_2_ at 37 °C.

### 2.6. Viruses

The influenza viruses used in this study are listed in Table 2. Viruses were passaged in MDCK cells at 35 °C following WHO-recommended protocols [27,28]. Viral stocks were prepared in DMEM containing 0.5% bovine serum albumin (BSA) (Invitrogen) and stored as single-use aliquots at −80 °C. Viral stock titers were quantified by hemagglutination and TCID_50_ assays. All influenza viruses used in this study were the cell culture-based candidate vaccine viruses (ccCVVs) recommended by the WHO for the appropriate seasons in the northern hemisphere. SARS-CoV-2 psuedovirus was obtained from SinoBiological (Beijing, China).

### 2.7. In Vitro Assays

In vitro activity of Luc-A_120_ and Luc-A_30_(T_2_A_30_)_3_ mRNA was measured in BHK-21 cells by luciferase assay and expressed as relative luminescence units (RLU). BHK-21 cells were seeded at 1 × 10^4^ cells per well in a 96-well cell culture treated plate in 100 μL growth media and incubated overnight at 37 °C, 5% CO_2_. The next day, cells were transfected in triplicate with 100 ng/well of Luc-A_120_ or Luc-A_30_(T_2_A_30_)_3_ mRNA using Lipofectamine MessengerMax (Invitrogen) according to the manufacturer’s instructions. Transfected cells were incubated for 24 h at 37 °C, 5% CO_2_ prior to luciferase activity assay. Luciferase activity was measured with Bio-Glo™ reagent (Promega, Madison, WI, USA) added to the transfected cells at a 1:1 volume ratio. Plates were incubated at room temperature in the dark on an orbital shaker for 15 min prior to reading on SpectraMax ID5 plate reader (Molecular Devices, San Jose, CA, USA).

### 2.8. In Vivo Immunogenicity Studies

All in vivo experiments were conducted in a vivarium operated by Mispro, Durham, NC, in accordance with the approved IACUC protocols. Naïve 6-to 8-week-old female BALB/c mice were sourced from The Jackson Laboratory (Bar Harbor, ME, USA). Mice were given food and water ad libitum throughout the course of all studies.

Luciferase activity in vivo was detected after intramuscular (I.M.) delivery of RTU LNP-formulated Luc-A_120_ or Luc-A_30_(T_2_A_30_)_3_ mRNA (5 µg per injection). In vivo imaging was performed 16 h after injection, using VivoGlo™ Luciferin (Promega) substrate on a Pearl Trilogy Imaging System (LI-COR Biosciences, Lincoln, NE, USA).

Mouse immunogenicity studies were performed with groups of 5 to 8 animals vaccinated on Day 0 (D0) and D21 with mRNA vaccines or licensed comparator vaccines: Fluad (CSL, Melbourne, Australia), Fluzone HD (Sanofi, Paris, France), and Spikevax (Moderna, MA, USA). Clinical signs and mouse weight were monitored throughout each experiment.

#### 2.8.1. Hemagglutination Inhibition (HAI) Assay

HAI titers were measured using serum samples collected on D14 and D35 (14 days after the prime and boost immunizations). HAI titer assays were performed in accordance with the WHO protocols [27,28]. In brief, serum was collected and treated with 3 parts receptor-destroying enzyme (RDE (II) “SEIKEN”, Denka, Tokyo, Japan), heat-inactivated, and diluted with 4 parts PBS. A two-fold dilution series of serum samples was prepared on 96-well plates and mixed with 4 HAU/25 μL of the appropriate influenza virus. After 1 h incubation, 50 μL of 0.5% turkey red blood cells (Lampire, Pipersville, PA, USA) in PBS (Invitrogen) were added, and the blood fall pattern was evaluated after 30 to 90 min.

#### 2.8.2. SARS-CoV-2 Pseudovirus Neutralization Assay

COVID-19 neutralizing antibody titers were measured in mouse sera using 293T-ACE2 cells (Vazyme) and SARS-CoV-2 Omicron XBB.1.5 pseudoviral particles (SinoBiological). 293T-ACE2 cells express hACE2 receptors and are permissive to infection by the S-protein pseudotyped viral particles. XBB.1.5 pseudotyped viral particles contain the luciferase reporter gene, permitting pseudovirus viral entry measurements via the luciferase activity assay. The pseudovirus was incubated with serially diluted mouse sera at 37 °C for 1 h before adding it to 3 × 10^4^ reporter cells plated in 96-well plate format. Equal 50 µL volumes of the cell suspension and pseudovirus/sera mixes were added into each well. The plates were centrifuged at 700 rpm for 15 min at 4 °C. The cells were cultured with pseudovirus and diluted sera for an additional 48 h. The 96-well plates were equilibrated to room temperature for 30 min. Then, 100 μL supernatant was removed from each well, and 100 μL/well Bio-Lite luciferase detection reagent (Bio-Lite Luciferase Assay System, Vazyme) was added. The plates were shaken for 3 min, and the luminescence was immediately measured by a microplate reader.

#### 2.8.3. COVID-19 IgG Titer Assay

Mouse sera were analyzed for RBD-specific antibody responses by ELISA. Briefly, 96-well ELISA plates were precoated overnight with 1 μg/mL of SARS-CoV-2 XBB.1.5 (Omicron) spike protein RBD (SinoBiological) in carbonate buffer pH 9.6 (Candor Bioscience, Wangen im Allgäu, Germany) at 4 °C and blocked with assay buffer (Invitrogen) for 1 h at 37 °C. The plates were washed three times with PBST. Serially diluted mouse sera (2-fold serum dilutions in PBS prepared in duplicate) were added to the plates and incubated at 37 °C for 2 h, followed by three washes. Bound antibodies were incubated with HRP-conjugated goat anti-mouse IgG (1:15,000, Jackson ImmunoResearch, West Grove, PA, USA # 115-035-003), anti-mouse IgG1 (1:12,000, ThermoFisher, Waltham, MA, USA, #PA1-74421) or anti-mouse IgG2a (1:5000, ThermoFisher, #A-10685) for 1 h at 37 °C. The enzymatic reaction was performed with ELISA TMB stabilized chromogen (Invitrogen) and stopped by ELISA stop solution (Invitrogen). The absorbance at 450 nm was measured by a Molecular Devices plate reader SpectraMax iD5. The endpoint titer was calculated as the highest dilution of the serum sample that produced a signal above the cut-off set as 2.1-fold the value of the blank.

### 2.9. Statistical Analysis

The assays were performed with duplicate technical replicates for all biological replicates. Statistical analysis was performed using GraphPad Prism (10) software. Differences in mean values among more than two groups were determined using ANOVA with Tukey’s test for mean comparisons. *p* < 0.05 (*), *p* < 0.01 (**), *p* < 0.001 (***), and *p* < 0.0001 (****) indicate statistically significant differences; not significant (ns).

## 3. Results

### 3.1. Vector Optimization

Segmented or heteromeric poly(A) tail sequences have been shown to increase plasmid stability and mRNA expression [29]. The Pfizer–BioNTech (BNT162b2) COVID-19 vaccine is an example of a commercial product incorporating a 10 nucleotide spacer inserted into the poly(A) sequence [30]. We developed a novel segmented poly(A) tail by minimizing the length of the non-A spacer placed between homomeric adenosine stretches. A homomeric tail length of approximately 120 A’s is considered optimal for maintaining plasmid stability and maximizing the translation efficiency of synthetic mRNAs, as longer tails do not significantly increase protein expression [31]. Our hybrid poly(A) sequence A_30_(T_2_A_30_)_3_ was tested against a homomeric poly(A) tail sequence of 120 adenosine residues (A_120_).

**Figure 2:**
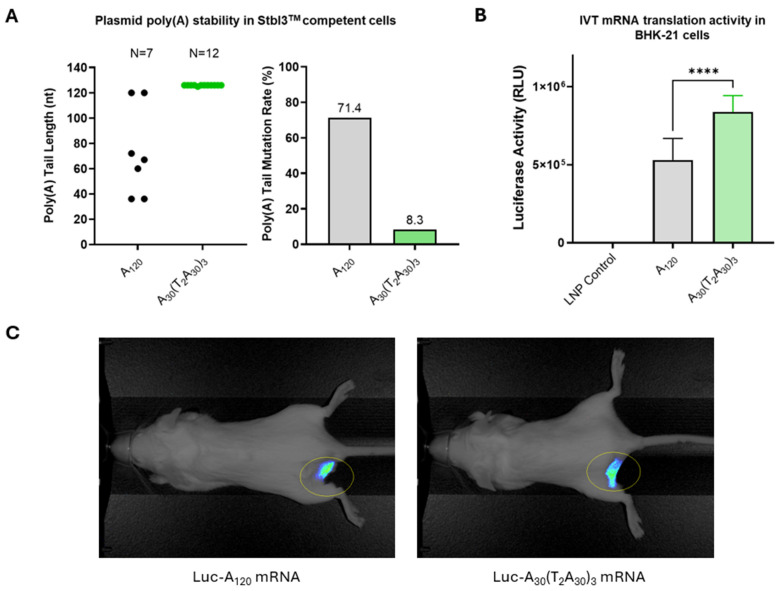
Vector optimization. (**A**) Improved stability of plasmids containing hybrid poly(A)-T_2_ sequence: *E. coli* Stbl3^TM^ cells were transformed with pLuc-A_120_ or pLuc-A_30_(T_2_A_30_)_3_ plasmids containing homopolymeric poly(A) sequence (A_120_) or hybrid poly(A)-T_2_ sequence (A_30_(T_2_A_30_)_3_). Poly(A) tail length was analyzed for individual bacterial clones. (**B**) In vitro activity of Luc-A_120_ and Luc-A_30_(T_2_A_30_)_3_ mRNA in transfected BHK-21 cells. Luciferase activity was determined in RLU (relative luminescence units). (**C**) In vivo imaging of Luc-A_120_ and Luc-A_30_(T_2_A_30_)_3_ mRNA expression following i.m. delivery in mice. *p* < 0.05 (*), *p* < 0.01 (**), *p* < 0.001 (***), and *p* < 0.0001 (****) indicate statistically significant differences; not significant (ns).

Plasmid stability was evaluated by transforming pLuc-A_120_ and pLuc-A_30_(T_2_A_30_)_3_ plasmids into *E. coli* Stbl3™ cells. Only two of seven pLuc-A_120_ plasmid transformed *E. coli* colonies contained A_120_ poly(A) tail, while eleven of twelve pLuc-A_30_(T_2_A_30_)_3_ plasmid transformed *E. coli* colonies contained the full-length A_30_(T_2_A_30_)_3_ hybrid poly(A) tail. The corresponding mutation rates of pLuc-A_120_ and pLuc-A_30_(T_2_A_30_)_3_ plasmids in transformed *E. coli* cells were 71.4% and 8.3%, respectively (Figure 2A). In addition, the only mutated pLuc-A_30_(T_2_A_30_)_3_ plasmid isolate had a single A nucleotide deletion, whereas the deletion mutations of poly(A) tails seen in pLuc-A_120_ plasmids ranged from 48 to 84 bases.

In vitro translation activities of mRNAs transcribed from the pLuc-A_120_ or pLuc-A_30_(T_2_A_30_)_3_ plasmids were measured by luciferase assay in BHK-21 cells. The cells transfected with Luc-A_30_(T_2_A_30_)_3_ mRNA had approximately 2-fold higher luciferase activity than those transfected with Luc-A_120_ mRNA (Figure 2B), suggesting that Luc-A_30_(T_2_A_30_)_3_ mRNA has improved translational activity compared with conventional Luc-A_120_ mRNA. Finally, robust in vivo luciferase expression was confirmed in mouse muscle tissue after injection of RTU LNP-formulated Luc-A_30_(T_2_A_30_)_3_ and Luc-A_120_ mRNA (Figure 2C).

### 3.2. Optimization of Influenza HA Antigen Design

The details of the influenza HA construct design can be found in Table S1. Briefly, throughout the manuscript, we adopted the terms monovalent, bivalent, and trivalent HA antigens based on the number of HA ectodomains encoded within a single open reading frame of an mRNA species. The monovalent HA antigens corresponded to full-length wild-type membrane HA proteins. The bivalent HA antigens comprised two distinct HA ectodomains joined by a T4 foldon domain flanked by Gly-rich flexible linkers (bivalent dumbbell design). In turn, the trivalent antigens included three HA ectodomains joined by two T4 foldon/flexible linker segments (trivalent dumbbell design). Different HA antigen combinations were always tested as components of quadrivalent or trivalent influenza vaccines. Thus, quadrivalent influenza mRNA vaccines comprised either four mRNAs encoding monovalent HA antigens or two mRNAs encoding bivalent HA antigens, while the trivalent mRNA vaccines included either a single mRNA encoding a trivalent HA antigen or two mRNAs encoding bivalent HA antigens, with one of the HA antigens encoded by both mRNA species. This approach allowed us to directly compare the performance of our mRNA vaccines to the strain-matched commercial quadrivalent or trivalent seasonal influenza vaccines (Fluad or Fluzone HD).

#### 3.2.1. Monovalent and Bivalent HA Antigens

Immunogenic properties of monovalent and bivalent HA antigens were compared by testing experimental mRNA vaccines formulated using a proprietary Immorna RTU LNP technology developed to ensure the long-term stability of the vaccines at 2–8 °C. The stability profile of the RTU LNP formulation is shown in Figures S2–S4.

**Figure 3:**
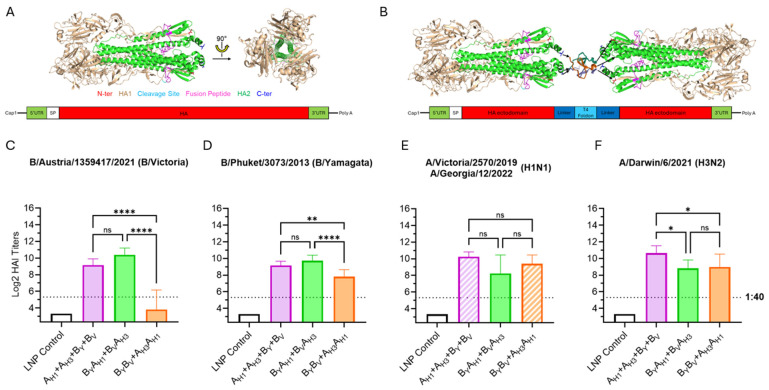
Bivalent HA antigen design and immunogenicity. (**A**) Ribbon model of the native trimeric form of the ectodomain of the influenza HA protein; and a graphic showing the mRNA design for monovalent HA antigens, which contain the full HA protein, including transmembrane and cytoplasmic domains. (**B**) Ribbon model of the bivalent antigen comprising two HA ectodomains linked by T4 foldon and forming a homotrimer and a graphic showing the mRNA encoding the secreted bivalent “dumbbell” HA. Ribbon models are conceptual visual aids describing theoretical structures based on published HA (PDB 5W6R) and T4 foldon (PDB 1RFO) structures. (**C**–**F**) Day 35 serum HAI geometric mean titers (GMT) against seasonal influenza viruses. Graph bars represent HAI GMTs of individual mouse sera (N = 6 per group): LNP Control (200 μL); A_H1_ + A_H3_ + B_V_ + B_Y_ vaccine (3 µg each mRNA) expressing monovalent HA antigens from influenza A(H1N1), A(H3N2), B-Victoria and B-Yamagata strains; B_Y_A_H1_+B_V_A_H3_ vaccine (6 µg each mRNA) expressing bivalent HA antigens with both HA dumbbells including one B- and one A- influenza strain-derived HA ectodomain (B/A design); B_Y_B_V_ + A_H3_A_H1_ vaccine (6 µg each mRNA) expressing bivalent HA antigens with the HA dumbbells including either two B- or two A-strain-derived HA ectodomains (B/B and A/A design). Striped graph bars in (**E**) indicate GMT elicited against matched 2022/23 (H1N1) seasonal influenza A-strain; the remaining data represent HAI titers against the 2023/24 seasonal strains. Summaries of HAI GMTs and seroconversion rates are included in Table S2A. *p* < 0.05 (*), *p* < 0.01 (**), *p* < 0.001 (***), and *p* < 0.0001 (****) indicate statistically significant differences; not significant (ns).

All mRNA vaccine groups expressed HA antigens for H1N1 and H3N2 influenza A subtypes and B/Victoria and B/Yamagata lineages, which were abbreviated to A_H1_, A_H3_, B_V_ and B_Y_, respectively. Monovalent and bivalent HA antigen structures can be seen in Figure 3A,B. Two bivalent HA antigen configurations, B_Y_A_H1_ + B_V_A_H3_ and B_Y_B_V_ + A_H3_A_H1_, were tested. As shown in Figure 3C–F, the most significant differences between the vaccine groups were observed in responses against influenza B-derived HA antigens. Particularly, the B_Y_B_V_ dumbbell antigen elicited low post-boost HAI geometric mean titers (GMTs) against B/Austria/1359417/2021 (below the 1:40 titer threshold). Considering the suboptimum performance of the B_Y_B_V_ dumbbell design, optimization of the B_Y_A_H1_ + B_V_A_H3_ vaccine configuration was chosen as the major direction for further vaccine development.

#### 3.2.2. Bivalent HA Antigen Optimization

We sought to improve the efficacy of the B_Y_A_H1_ + B_V_A_H3_ mRNA vaccine by optimizing the length of the Gly-rich linker, encoded by the bivalent dumbbell, between HA antigen ectodomains and by comparison of membrane-bound and secreted bivalent HA antigen formats. Two types of flexible Gly-rich linkers consisting of either seven amino acid residues (used in the previous experiments) or thirty-seven amino acid residues (extended linker) were inserted between the T4 foldon domain and the second (downstream) HA ectodomain. Membrane-bound and secreted variants of bivalent dumbbell HA antigens were formed with and without the native transmembrane and cytoplasmic HA domains of the downstream HA antigens, respectively. Both membrane-bound and secreted variants of bivalent dumbbell HA antigens with either a short or extended Gly-rich linker were studied. All tested vaccine candidates, including Fluad, contained HA antigens derived from all four 2023–2024 seasonal influenza strains. The vaccine doses for Fluad (50 µL) and mRNA vaccines (5 µg total RNA per immunization) were selected to match 1/10 of the intended human dose.

**Figure 4:**
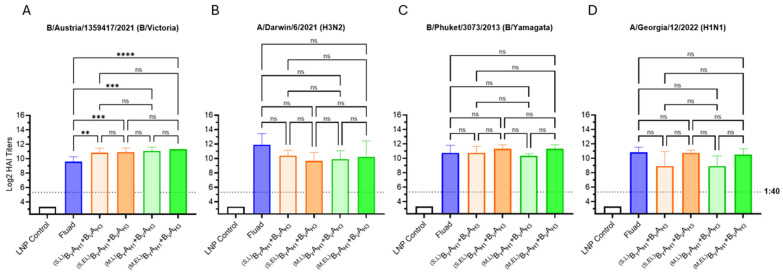
Optimization of bivalent HA antigen design. (**A**–**D**) Day 35 serum HAI GMTs (N = 6 per group) against 2023/24 seasonal influenza viruses were determined for LNP Control (200 μL); Fluad (50 μL) and B_V_A_H3_ + B_y_A_H1_ vaccines (5 μg each mRNA) comprising B/A dumbbell HA antigens expressed either as fusion proteins connected through a linker or an extended linker (L or EL), and/or in a secreted or membrane-bound (S or M) antigen format. Summaries of HAI GMTs and seroconversion rates are included in Table S2B. *p* < 0.05 (*), *p* < 0.01 (**), *p* < 0.001 (***), and *p* < 0.0001 (****) indicate statistically significant differences; not significant (ns).

All variants of the bivalent mRNA vaccines performed comparably and elicited HAI titers higher than (B/Victoria, Figure 4A) or similar (H1N1, H3N2, and B/Yamagata, Figure 4B–D) to the commercial comparator vaccine Fluad (Figure 4). We were unable to conclusively demonstrate whether the extension of the Gly-rich linker downstream of T4 foldon enhanced immunogenicity of the bivalent dumbbell HA antigens as the differences in HAI GMTs between short versus extended linker vaccine groups were not statistically significant. However, we saw consistently higher mean HAI titers for the extended linker B_Y_A_H1_ antigens against both B/Yamagata and H1N1 strains (Figure 4C,D), expressed either as membrane-bound or secreted proteins and the extended linker was used in subsequent studies.

#### 3.2.3. Trivalent HA Antigens

**Figure 5:**
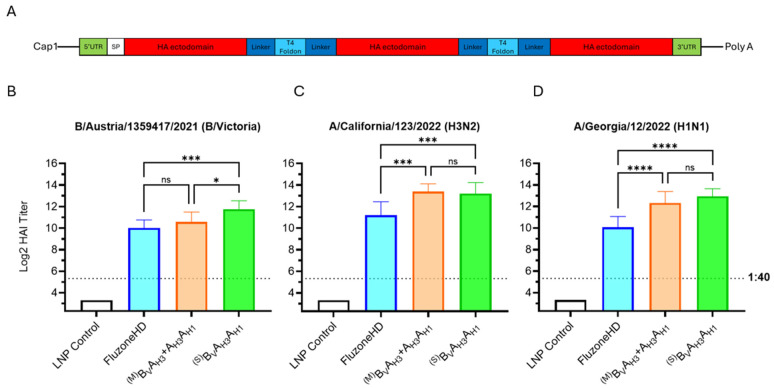
Bi- and trivalent dumbbell antigen designs. (**A**) Graphic showing the mRNA design for trivalent HA antigens. (**B**–**D**) Day 35 serum HAI GMTs (N = 8 per group) against 2024/25 seasonal influenza viruses were determined for LNP Control (200 μL); Fluzone HD (50 μL); B_V_A_H3_ + A_H3_A_H1_ influenza vaccines expressing membrane (M) B/A and A/A HA bivalent dumbbells (5 μg each); B_V_A_H3_A_H1_ mRNA vaccines (10 μg) expressing secreted (S) trivalent HA dumbbells. Summaries of HAI GMTs and seroconversion rates are included in *p* < 0.05 (*), *p* < 0.01 (**), *p* < 0.001 (***), and *p* < 0.0001 (****) indicate statistically significant differences; not significant (ns).

For the 2024–2025 season, the WHO and FDA recommended that manufacturers switch to a trivalent influenza vaccine format, excluding the B/Yamagata strain, which had not been detected in circulation since 2020. While the bivalent HA dumbbell design is clearly compatible with a quadrivalent vaccine, creating a bivalent HA dumbbell design that encodes three antigens for a trivalent vaccine is less straightforward. To this end, we tested trivalent mRNA vaccines containing two antigen formats: (1) A bivalent HA dumbbell mRNA vaccine (B_V_A_H3_ + A_H3_A_H1_) where the A_H3_ antigen is encoded by both mRNA species and (2) A trivalent HA dumbbell mRNA vaccine (B_V_A_H3_A_H1_) in which a single mRNA species encodes all three HA antigens (design seen in Figure 5A). We selected the A_H3_ antigen to be included in both mRNA species for the following reasons: (1) In the previous experiment (Figure 4), the HAI GMT induced against A/Darwin/6/2021 (H3N2) by the membrane-bound extended linker vaccine group was lower than the HAI GMTs induced against B/Austria/1359417/2021 (B/Victoria) or A/Georgia/12/2022 (H1N1), while the HAI GMT induced against A/Darwin/6/2021 (H3N2) by the commercial comparator vaccine Fluad was higher than the HAI GMTs induced against B/Austria/1359417/2021 (B/Victoria) or A/Georgia/12/2022 (H1N1) and (2) based on our own data (Figure 3 and Figure 4) we knew that the A_H3_ antigen would be immunogenic both when encoded downstream of the B_V_ or upstream of the A_H1_ antigen. The bivalent HA dumbbells were expressed as membrane proteins, while the trivalent HA dumbbell antigen was expressed as a secreted protein to reduce the risk of limited accessibility to the membrane-proximal HA ectodomains by B-cell receptors.

The experimental trivalent seasonal influenza mRNA vaccines were evaluated against the 2024/25 trivalent Fluzone HD vaccine, as the 2024/25 Fluad vaccine was not commercially distributed at the time the in vivo experiments were performed (Figure 5B–D). The trivalent HA dumbbell vaccine group (B_V_A_H3_A_H1_) significantly outperformed Fluzone HD across all three seasonal strains, but while the HAI GMTs of the bivalent HA dumbbell vaccine group (B_V_A_H3_ + A_H3_A_H1_) were higher than Fluzone HD, only those against A/California/123/2022 and A/Georgia/12/2022 were statistically significant (Figure 5B–D). Due to the robust performance of the mRNA vaccine encoding the trivalent B_V_A_H3_A_H1_ HA antigen, the single mRNA species encoded trivalent HA dumbbell vaccine design was selected as the lead candidate for further studies.

### 3.3. Application of Dumbbell Antigens to Influenza/COVID-19 Combination mRNA Vaccines

Since we have previously developed a dumbbell COVID-19 mRNA vaccine that demonstrated strong immunogenicity in the recently completed Phase 1 clinical trial (NCT05743335) [32], we decided to evaluate the performance of a combination vaccine including dumbbell antigens from both seasonal influenza and SARS-CoV-2 viruses. To this end, a dumbbell COV_XBB_COV_XBB_ containing two SARS-CoV-2 Omicron XBB.1.5 variant S-protein RBD antigens, connected by a T4 foldon domain and Gly-rich linkers, was constructed. The details of the protein and mRNA design of this vaccine are shown in Table S1.

To perform a direct comparison of our experimental influenza/COVID-19 combination vaccine with Spikevax, a commercial LNP mRNA COVID-19 vaccine, B_V_A_H3_A_H1,_ and COV_XBB_COV_XBB_ mRNAs were mixed and formulated as a traditional encapsulated LNP mRNA vaccine rather than an RTU LNP mRNA vaccine evaluated in previously described studies. The combination vaccine was tested at low and high doses (3 and 12 µg total mRNA per immunization, respectively) corresponding to 1/10th of the target human vaccine dose in the 30 to 120 µg range.

**Figure 6:**
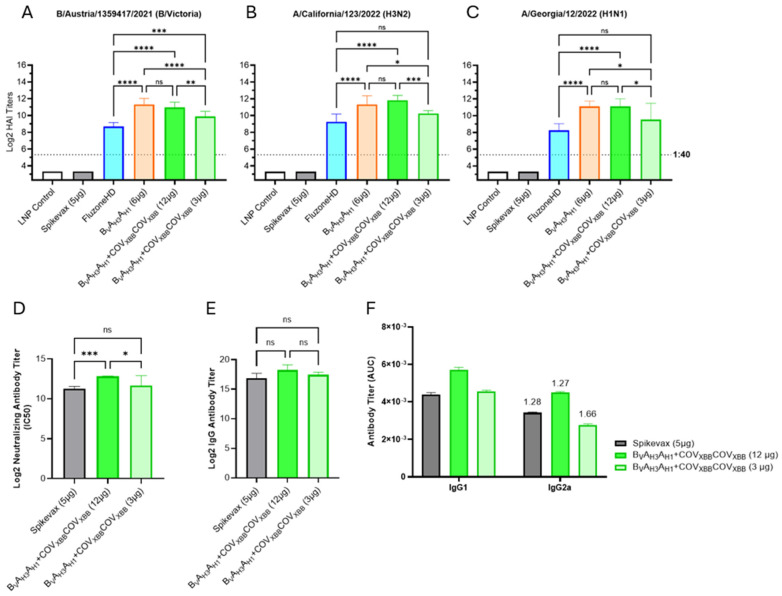
Encapsulated LNP-formulated influenza/COVID-19 combination mRNA vaccine. (**A**–**C**) Day 35 serum HAI GMTs (N = 7 per group) against 2024/25 seasonal influenza viruses were determined for LNP Control (200 μL); Spikevax (50 μL); Fluzone HD (50 μL); B_V_A_H3_A_H1_ tested as an influenza-only, or combination vaccine with COV_XBB_COV_XBB_ mRNA tested at low and high doses (3 μg or 12 μg total mRNA, with a 1:1 mRNA weight ratio of two mRNA species). (**D**) Average neutralizing antibody titers against SARS-CoV-2 Omicron XBB.1.5 pseudovirus were evaluated in mouse sera of the following vaccination groups: Spikevax (50 μL) and B_V_A_H3_A_H1_ and COV_XBB_COV_XBB_ mRNA combination vaccine tested both at low and high doses (3 μg or 12 μg total mRNA). Summaries of HAI GMTs and seroconversion rates are included in Table S2D. (**E**) Average total RBD-specific IgG titers of Day 35 mouse sera were tested for the following vaccine groups: Spikevax (50 μL); B_V_A_H3_A_H1_ with COV_XBB_COV_XBB_ in both a high and low dose (12 μg or 3 μg total, respectively). (**F**) Average total RBD-specific IgG1 and IgG2a titers of pooled D35 mouse serum. Numbers above IgG2a bars indicate IgG1/IgG2a ratio. *p* < 0.05 (*), *p* < 0.01 (**), *p* < 0.001 (***), and *p* < 0.0001 (****) indicate statistically significant differences; not significant (ns).

The encapsulated LNP mRNA vaccines, expressing B_V_A_H3_A_H1_ HA antigen alone or in combination with the COV_XBB_COV_XBB_ dumbbell, elicited strong HAI titers against all three influenza HA antigens (Figure 6A–C). Similarly to the RTU LNP-formulated B_V_A_H3_A_H1_ mRNA vaccine (Figure 5), the encapsulated LNP mRNA vaccines, including 6 µg of B_V_A_H3_A_H1_ mRNA (influenza-only vaccine as well as the high dose combination vaccine), significantly outperformed Fluzone HD across all three seasonal strains (Figure 6A–C). Importantly, we saw no interference between the COV_XBB_COV_XBB_ mRNA and the B_V_A_H3_A_H1_ mRNA co-formulated at a 1:1 weight ratio since the influenza-only vaccine and the combination influenza/COVID-19 vaccine elicited comparable HAI GMTs. Finally, the low dose combination mRNA vaccine group (containing 1.5 μg of B_V_A_H3_A_H1_ mRNA and 1.5 μg of COV_XBB_COV_XBB_ mRNA) also outperformed Fluzone HD, although while the HAI GMT differences were significant against B/Austria/1359417/2021, the differences against the seasonal influenza A-strains were not. A comparison between HAI GMTs of encapsulated LNP- and RTU LNP-formulated vaccines expressing B_V_A_H3_A_H1_ HA antigen in combination with the COV_XBB_COV_XBB_ dumbbell at high and low doses can be seen in Figure S5.

To assess COVID-19 responses, neutralizing antibody titers were measured for individual mouse sera using a SARS-CoV-2 pseudovirus neutralization assay performed on HEK-293 ACE2 cells (Figure 6D). Significantly higher neutralizing antibody titers were induced in mice immunized with the high dose combination mRNA vaccine (containing 6 µg of COV_XBB_COV_XBB_ mRNA) than with Spikevax (5 µg mRNA per dose, Figure 6D), while the titers elicited by the low dose combination vaccine (including 1.5 µg of COV_XBB_COV_XBB_ mRNA) were comparable to Spikevax. The anti-RBD total IgG titer results (Figure 6E, F) correlated very well with the neutralizing antibody titers; however, the titer differences were not statistically significant. Finally, the IgG1 to IgG2a titer ratios were similar between Spikevax and the combination mRNA vaccines, suggesting balanced Th1 and Th2 response profiles induced by both vaccines (Figure 6F) [33].

### 3.4. Single mRNA Encoded Tetravalent Influenza/COVID-19 Combination Vaccines

**Figure 7:**
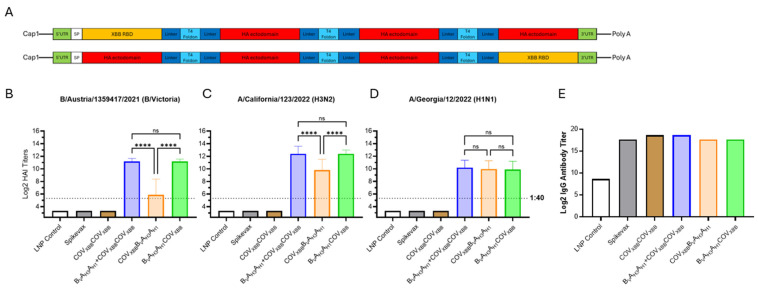
Tri- and tetravalent dumbbell antigen designs for combination influenza/COVID-19 mRNA vaccine. (**A**) Graphic showing the mRNA design for tetravalent chimeric HA and XBB antigen dumbbells. (**B**–**D**) Day 35 serum HAI GMTs (N = 7 per group) against 2024/25 seasonal influenza viruses were determined for LNP Control (200 μL); Spikevax (50 μL); COV_XBB_COV_XBB_ (6 μg mRNA); B_V_A_H3_A_H1_ tested in combination vaccine with COV_XBB_COV_XBB_ (12 μg total mRNA, 6 μg each); COV_XBB_B_V_A_H3_A_H1_ mRNA vaccine (12 μg) and B_V_A_H3_A_H1_COV_XBB_ mRNA vaccine (12 μg) expressing secreted tetravalent chimeric dumbbell antigens. Summaries of HAI GMTs and seroconversion rates are included in Table S2E. (**E**) Average total RBD-specific IgG titers of pooled D35 mouse sera were determined for the same experimental groups. *p* < 0.05 (*), *p* < 0.01 (**), *p* < 0.001 (***), and *p* < 0.0001 (****) indicate statistically significant differences; not significant (ns).

Finally, we constructed two variants of a tetravalent antigen comprising three influenza HA ectodomains and the SARS-CoV-2 S-protein RBD. Each of the chimeric antigens were encoded by a single mRNA species. The antigen details can be seen in Table S1. The COV_XBB_ RBD antigen was linked to the N- or C-terminus of the B_V_A_H3_A_H1_ trivalent HA antigen via a T4 foldon/extended Gly-rich linker sequences to produce the tetravalent chimeric fusion protein (Figure 7A).

The experimental tetravalent chimeric mRNA vaccines were compared with the trivalent B_V_A_H3_A_H1_ mRNA vaccine for vaccine efficacy against influenza HA antigens. The position of the COV_XBB_ antigen within the tetravalent dumbbell dramatically affected influenza HAI GMTs of the tetravalent mRNA vaccine. When the COV_XBB_ antigen was linked with the N-terminus of the B_V_A_H3_A_H1_ antigen, the induction of humoral responses against the B_V_ and A_H3_ HA antigens, but not the A_H1_ HA antigen, were significantly lower than those against the trivalent B_V_A_H3_A_H1_ dumbbell HA antigen (Figure 7B–D). However, when the COV_XBB_ antigen was linked to the C-terminus of the B_V_A_H3_A_H1_ antigen, HAI GMTs against all three HA antigens were comparable to the titers elicited by the trivalent B_V_A_H3_A_H1_ HA dumbbell (Figure 7B–D).

The total IgG titer against the XBB.1.5 S-protein RBD were determined for pooled serum samples (Figure 7E). The IgG titers were two-fold higher for both the COV_XBB_COV_XBB_ and B_V_A_H3_A_H1_ + COV_XBB_COV_XB_ mRNA vaccines compared with Spikevax. Interestingly, both tetravalent chimeric dumbbell groups (COV_XBB_ B_V_A_H3_A_H1_ and B_V_A_H3_A_H1_COV_XBB_) had comparable total IgG titers to those of Spikevax, further suggesting high potency of the multivalent mRNA vaccine design described in this report.

## 4. Discussion

A common problem for mRNA technologies is ensuring the optimum length and uniformity of poly(A) tail sequences. Native poly(A) sequences containing homomeric A stretches pose considerable manufacturing risks because of plasmid instability. While mRNA molecules with longer poly(A) tails have increased stability and expression within cells, the longer homomeric poly(A) tail sequences are harder to maintain in plasmids [29]. Heteromeric poly(A) tail sequences were developed to enhance plasmid stability and to improve mRNA expression. For example, the BNT162b COVID-19 vaccine has a composite poly(A) tail, with 30 As followed by a 10 nucleotide spacer (5′-GCAUAUGACU-3′) and then 70 additional A nucleotides [30]. We developed a new heteromeric poly(A) sequence, referred to as hybrid poly(A), with the non-A spacer minimized to two nucleotides: T_2_ in the plasmid template transcribed into U_2_ or Ψ_2_ in mRNA or N1-methylpseudouridine modified mRNA. We also minimized the length of the individual homomeric A sections to 30 As, in line with the model of consecutive poly(A)-binding protein (PABP) molecules bound to the same poly(A) tract as a series of repeating units covering approximately 27 As (reviewed in [34]). Our hybrid poly(A) sequence is functional both in terms of improved plasmid stability and efficient expression in vitro and in vivo.

It is worth noting that the bi-, tri-, and tetravalent antigen designs described in this study present additional plasmid stability risks because of their inherent sequence complexity. The inclusion of multiple Gly-rich linker regions, T4 foldon domains, and two or three HA antigens within the same sequence increases the recombination risk between direct repeats of identical or highly homologous sequences [35]. The Gly-rich regions additionally present cloning challenges because of the high GC content of consecutive Gly codons. While we have not observed plasmid instability of the plasmids described in this study, the use of the novel hybrid poly(A) sequence reduced the bacterial colony screening by 5-fold during the plasmid cloning (Figure 2A).

In this report, we describe a novel HA antigen design primarily developed in order to optimize anti-influenza B virus responses. Initially, we prepared a quadrivalent mRNA vaccine consisting of two mRNA species, with one containing two type A-strain HA antigens (A_H3_A_H1_ dumbbell) and the other containing two type B lineage HA antigens (B_Y_B_V_ dumbbell). Expression of influenza type A HA antigens from one mRNA and influenza type B HA antigens from another mRNA would allow us to adjust the mRNA ratio, thereby controlling the relative expression of and immune responses against influenza A and B HA antigens. However, the immune responses elicited by the B/B dumbbell were dramatically lower compared with the groups immunized with the monovalent mRNAs encoding wild-type HA antigens. Particularly within the B/B dumbbells, the HA ectodomain in the second position was significantly less immunogenic than the HA ectodomain in the first position. Initially, we speculated that the T4 foldon functions more efficiently as a C-terminal trimerization domain attached to the upstream HA ectodomain as opposed to the N-terminal trimerization domain fused to the downstream HA ectodomain. Consequently, the differences in immunogenicity among the downstream HA antigens would directly reflect their intrinsic trimerization activity. In this respect, our immunogenicity data shown in Figure 3 are aligned with the trimerization potential of B < H1 (Group 1) < H3 (Group 2) HA ectodomains [20,36,37,38,39]. However, this hypothesis does not explain the low immunogenicity of the B HA antigen expressed as an internal domain of the COV_XBB_B_v_A_H3_A_H1_ tetravalent vaccine (Figure 7). In this case, both the upstream and downstream T4 foldon domains could contribute to the trimerization of the B_V_ HA ectodomain. An alternative hypothesis is that the fusion of an upstream sequence, in this case, either the short or extended linker, affects the trimerization of the downstream type B HA antigen. It is intriguing to speculate either that the processing of the native N-terminal sequences of B influenza HA domains and/or, potentially, the presence of the free N-terminus generated after the signal peptidase cleavage may be critical to the proper B influenza HA folding and trimerization.

Based on the initial experiments pointing to the preferred upstream position for B influenza-derived HA ectodomains, we decided to shift to the B/A dumbbell HA antigens. Simultaneously, we evaluated the immunogenicity of dumbbells with short and extended Gly-rich flexible linkers between the T4 foldon and a downstream HA ectodomain. The extended 37 amino acid long linkers were introduced to minimize the risk of steric hindrance potentially interfering with the trimerization of the downstream antigens in the B/A dumbbell structure. Both design features were tested in the context of secreted and membrane-bound bivalent antigens. The subsequent mouse immunogenicity study (Figure 4) demonstrated improved performance of B/Victoria and B/Yamagata HA antigens expressed in a B/A dumbbell format with both type B antigens eliciting high HAI GMTs and with titers against B/Austria/1359417/2021 significantly exceeding the titers induced by Fluad. The mRNA vaccine candidates with the extended Gly-rich flexible linkers had improved HAI GMTs for both antigens in the B_Y_A_H1_ dumbbell compared with their short linker counterparts, although the titer differences did not reach statistical significance.

The multivalent antigen “scaffold” based on the T4 foldon domains/extended Gly-rich flexible linkers was used to create bi, tri-, and tetravalent HA antigens, including chimeric dumbbells comprising both influenza and COVID-19 antigens. Therefore, we were able to develop a design compatible with the multidomain antigens with multiple trimerizing subunits, e.g., three T4 foldon domains and three HA ectodomains in B_v_A_H3_A_H1_COV_XBB_ tetravalent antigen (Figure 7). The mRNA-expressed mono-, bi-, and trivalent HA antigens elicit similar patterns of humoral immune responses to the commercial comparator Fluzone HD, an inactivated influenza virus vaccine, as evidenced by similar trends in HAI, total anti-HA IgG, IgG1, and IgG2a antibodies, although the magnitude of the immune responses is higher for the mRNA encoded multivalent HA antigens. A representative data set illustrating anti-B_V_ HA protein responses is included in Supplemental Figure S1. We found that repeated T4 foldon domains encoded by a single polypeptide worked well in the context of the complex secreted fusion proteins. We consistently observed robust expression of dumbbell antigens that, during in vitro transfection studies, reached 500–2000 ng of protein expressed from 1000 ng of mRNA 24–48 h post-transfection. We also confirmed the expected molecular weight for both bi- and trivalent HA antigens Figure S6.

Moreover, this antigen design was also compatible with the membrane variants of the HA dumbbells, indicating that the trimerization mechanisms of membrane HA, which also involve HA transmembrane and cytoplasmic domains, are not disrupted by trimerization driven by the heterologous T4 foldon domains.

While both the secreted trivalent dumbbell and the membrane-bound bivalent dumbbell vaccines consistently outperform the HAI GMTs elicited by the licensed vaccines Fluad and Fluzone HD against the B/Victoria lineage (Figure 4, Figure 5 and Figure 6), the trivalent vaccine (B_v_A_H3_A_H1_) had 2-fold higher HAI GMTs against B/Austria/1359417/2021 than the bivalent vaccine (B_v_A_H3_ and A_H3_A_H1_) (Figure 5). The improved performance of the trivalent dumbbell antigen against influenza B offers an optimal solution for the historically low influenza B responses while decreasing the vaccine manufacturing complexity by including HA antigens from all seasonal influenza strains on a single mRNA. Recently, Moderna reported that their mRNA-1083 vaccine (comprising 50 µg of Moderna mRNA-1010 vaccine candidate for seasonal influenza and mRNA-1283, the next-generation COVID-19 vaccine candidate) elicited statistically significantly higher immune responses than Fluzone HD against three seasonal influenza virus strains (H1N1, H3N2, and B/Victoria) in a 65 years and older cohort [7]. Although mRNA-1083 outperformed Fluzone HD, the HAI GMT ratios of the mRNA-1083 group versus Fluzone HD group were 1.16 for A(H1N1), 1.06 for A(H3N2) and 1.12 for B-Victoria. For comparison, the analogous titer ratios of our high dose encapsulated LNP B_V_A_H3_A_H1_ + COV_XBB_COV_XBB_ combination mRNA vaccine group versus Fluzone HD comparator were 7.26 for A(H1N1), 5.94 for A(H3N2) and 4.86 for B-Victoria, i.e., approximately 5 times greater these reported by Moderna for mRNA-1083. Our preclinical results were generated in a mouse model, with the high dose of LNP combination mRNA vaccine (6 µg of HA mRNA) designed to contain 1/10th the planned human dose or 60 µg of HA mRNA, which is approximately equivalent to the mRNA-1083 dose used by Moderna in human clinical trials.

Our lead influenza antigen designs were tested as individual vaccines or in combination with the COVID-19 mRNA vaccine expressing SARS-CoV-2 XBB.1.5 S-protein RBD antigen. Our RBD IgG and pseudovirus neutralization titers were higher than the titers induced by the commercial competitor Spikevax. While we saw excellent results for our COVID-19 dumbbells in our previous studies [32], here we were able to both replicate those results and verify mutual compatibility of the lead influenza and COVID-19 mRNA candidates to form a combination vaccine: we saw no significant interference of co-formulation and co-administration of the mRNAs included in the combination vaccine.

## 5. Conclusions

In summary, we developed a novel, highly immunogenic mRNA vaccine against seasonal influenza by combining vector improvements and developing bi- and trivalent dumbbell HA antigen designs. Expression of multiple antigens from a single mRNA minimizes the number of mRNA species included in the combination vaccines, thus greatly simplifying their manufacturing and release testing. The vectors and antigen designs described here are compatible with different mRNA formulations, including RTU LNP formulations with improved thermal stability, aiding in storage and distribution logistics in historically underserved areas. Our modular vaccine design is highly flexible and, conceptually, can be directly applied to additional trimeric viral antigens such as the HIV-1 envelope glycoprotein (Env) trimer, made up of gp120 and gp41 subunits, or the RSV F protein.

## Figures and Tables

**Table 1:** Vaccine antigens.

Virus	Strain	Isolate ID	Protein ID
Influenza	A/Wisconsin/588/2019 (H1N1)	EPI_ISL_404460	EPI1661231
Influenza	A/Wisconsin/67/2022 (H1N1)	EPI_ISL_15928563	EPI2224978
Influenza	A/Darwin/6/2021 (H3N2)	EPI_ISL_2233238	EPI1859992
Influenza	A/Massachusetts/18/2022 (H3N2)	EPI_ISL_16968012	EPI2413620
Influenza	B/Austria/1359417/2021 (B/Victoria)	EPI_ISL_2378894	EPI1868375
Influenza	B/Phuket/3073/2013 (B/Yamagata)	EPI_ISL_161843	EPI529345
SARS-CoV-2	hCoV-19/USA/RI-CDC-2-6647173/2022	EPI_ISL_16134259	GenBank: WAR32688.1

**Table 2:** Viral strains used for titer determination.

Virus	Supplier	Cat#	Strain
Influenza	NIBSC	21/346	A/Victoria/2570/2019 (H1N1)
Influenza	CDC	N/A	A/Georgia/12/2022 (H1N1)
Influenza	NIBSC	21/212	A/Darwin/6/2021 (H3N2)
Influenza	CDC	N/A	A/California/123/2022 (H3N2)
Influenza	NIBSC	21/224	B/Austria/1359417/2021 (B/Victoria)
Influenza	NIBSC	21/132	B/Phuket/3073/2013 (B/Yamagata)
SARS-CoV-2 S-protein pseudotyped lentivirus	SinoBiological	PSV030	SARS-CoV-2 B.1.1.529 sublineage XBB.1.5 (Omicron) Spike Pseudovirus

## Data Availability

Data presented in this study are available on request from the corresponding author.

## Supplementary Materials

The following supporting information can be downloaded at: https://www.mdpi.com/article/10.3390/vaccines13060628/s1, Supplemental Materials and Methods; Supplemental Tables and Figures: Table S1: Antigen composition, Table S2A–E. Tabulated log2 HAI GMTs, Table S3A,B. Microfluidic mixing for LNP preparation and buffer exchange and concentration of LNPs: key process parameters, Figure S1: IgG and HAI titers, Figures S2–S4: Release and stability testing data for RTU LNP formulation, Figure S5: Encapsulated LNP vs. RTU LNP formulation, Figure S6. Western blot analysis of bivalent and trivalent HA dumbbell antigens; Supplemental Information: Nucleotide Sequence of B_V_A_H3_A_H1_ mRNA, Amino Acid Sequence of B_V_A_H3_A_H1_ Antigen.

## Abbreviations

The following abbreviations are used in this manuscript:

COVID-19	coronavirus disease 2019
UTR	untranslated region
HAI	hemagglutination inhibition
RBD	receptor binding domain
IVT	in vitro transcription
LNP	lipid nanoparticle
RTU LNP	ready-to-use lipid nanoparticle
GMT	geometric mean titer
ccCVVs	cell culture-based candidate vaccine viruses
NIBSC	National Institute for Biological Standards and Control
CDC	Centers for Disease Control and Prevention
RLU	relative luminescence units
TCID_50_	Tissue Culture Infectious Dose 50%
