# Supplementary material for: Development of an Influenza/COVID-19 Combination mRNA Vaccine Containing a Novel Multivalent Antigen Design That Enhances Immunogenicity of Influenza Virus B Hemagglutinins
Source: Vaccines (Basel). 2025 Jun 11;13(6):628. doi: 10.3390/vaccines13060628 (PMC12197711; doi:10.3390/vaccines13060628)
Supplement: Supplementary file 1 [file vaccines-13-00628-s001.zip › vaccines-3637900-supplementary.pdf]

Supplemental Materials and Methods

Particle Size and Polydispersity Index (PDI)

Nano particle size analyzer (Zetasizer Ultra Red, Malvern Panalytical, Worcestershire, UK) measurements based on dynamic light scattering were used for the particle size and polydispersity index determination. mRNA was diluted with formulation buffer (10 mmol/L citrate buffer, 3% (w/v) Sucrose, pH 5.5) at volumetric ratios of 1:14-1:30 prior to testing. After equilibration to 25℃ for 5 min, the measurements were made using 173° scatter angle. The refractive index was set at 1.3310 and the viscosity at 0.890 cp. Measurements were performed in triplicates. The acceptance criterion for particle size results was the relative standard deviation (% RSD) of N=3 replicate measurements ≤ 10%.

mRNA Content

A commercial kit (Quant-iT™ RiboGreen kit, Invitrogen, R11490) was used to determine the RNA concentration according to the manufacturer's instructions. Measurements were performed in triplicates. The acceptance criterion for RNA concentration results was% RSD of N=3 replicate measurements ≤ 10%.

mRNA absorption efficiency determination by Triton X-100 treatment and RiboGreen quantification of total and free RNA

To quantify the total RNA concentration in LNP preparations, the samples were diluted with 1x Tris-EDTA (TE) buffer containing 1% Triton X-100 to obtain a final concentration within the RNA standard range (188 to 3000 μg/mL). To quantify the concentration of free RNA which had not been absorbed into LNP, the samples were diluted the with 1x Tris-EDTA (TE) buffer without Triton X-100. For each sample, RNA concentrations of total and free RNA were determined in triplicates using Quant-iT™ RiboGreen kit. The absorption efficiency was calculated as: 100%*([total RNA]-[free RNA]/[total RNA]). The acceptance criterion for results was% RSD of N=3 replicate determinations ≤ 10%.

Purity

mRNA was released from LNP preparations by Triton X-100 added to the final concentration of 1% (v/v). mRNA was then precipitated with LiCl, washed by adding chilled 70% ethanol, dried and diluted to a final concentration of 100 ng/μL. mRNA aliquots were heated at 70℃ for 2 min and cooled to 4℃ to denature RNA. The samples including 2 μL of denatured RNA (RNA quantity: 200 ng) and 22 μL RNA Diluent Marker (Agilent, Cat No. DNF-369-0004) were prepared in duplicates and subjected to capillary electrophoresis using Agilent 5300 Fragment Analyzer (Agilent Technologies, Santa Clara, California, USA). The acceptance criterion for results was % RSD of N=2 replicate purity determinations ≤ 10%.

In vitro potency

The BHK-21 cells were seeded into a 12-well plate at 12x10^4 cells per well and cultured at 37°C and 5% CO2 overnight. Cells were transfected with 200 μL mixture of mRNA-Lipofectamine 3000 (Invitrogen, Cat. No. L3000001) per well, in duplicates. Transfected cells were cultured for another 42 hours, and the cell supernatants were collected to measure the spike protein RBD by ELISA (SARS-CoV-2（2019-nCoV）Spike RBD ELISA Kit, Sino Biological, Cat. No. KIT40592). Measurements were performed in duplicates. The relative potency results were reported against the reference standard mRNA lot. The acceptance criterion for results was % RSD of N=2 relative potency determinations ≤ 30%.

Zeta potential determination by phase analysis light scattering

Zeta potential measurements of LNP dispersion were performed using Zetasizer Ultra Red, Malvern Panalytical, based on phase analysis light scattering. LNP samples were diluted with formulation buffer (10 mmol/L citrate buffer, 3% (w/v) Sucrose, pH 5.5) at volumetric ratios of 1:14-1:30 prior to testing. After equilibration to 25°C for 5 min, the measurements were run in triplicates, each with 10 to 100 runs, with 60 second delay between measurements. The acceptance criterion for results was %RSD of N=3 replicate measurements ≤ 5%.

Ionizable lipid concentration

An ultra-performance liquid chromatographic system (UPLC, H-class, Waters Corporation, Milford, Massachusetts, USA) with the evaporative light scattering detector was used to perform the LNP lipid analysis. Key parameters: Mobile phase gradient with Solution A (0.05% trifluoroacetic acid in water) and Solution B (0.05% trifluoroacetic acid in iso-propanol); Column: ACQUITY UPLC BEH C18, 130Å, 1.7μm, 2.1 mm ×50 mm, Waters. Cat. No. 186002350; Flow rate: 0.5 mL/min; Column temperature: 70°C; Injection volume: 2 μL; Drift tube temperature: 55°C; Nebulizer temperature: 40°C; Ionizable lipid standard range: 250-1000 μg/mL. The concentration of ionizable lipid was calculated against the standard curve. Measurements were performed in duplicates. The acceptance criterion for results was % RSD of N=2 replicate measurements ≤ 5%.

HA IgG Titer Assay

Mouse sera were analyzed for HA-specific antibody responses by ELISA. Briefly, 96-well ELISA plates were precoated overnight with 1 μg/mL of Influenza B/Austria/1359417/2021 HA protein (NIBSC, #21/316) in carbonate buffer pH 9.6 (Candor Bioscience, Wangen im Allgäu, Germany) at 4 °C and blocked with assay buffer (Invitrogen) for 1 hr at 37 °C. Plates were washed three times with PBST. Serial 2-fold dilutions of mouse sera in PBS were prepared in duplicates. The serum samples were added to the plates and incubated at 37 °C for 2 hrs, followed by three washes. Bound antibodies were incubated with HRP-conjugated goat anti-mouse IgG

(1:15000, Jackson ImmunoResearch, West Grove, PA, USA # 115-035-003), anti-mouse IgG1 (1:12000, ThermoFisher, Waltham, MA, USA, #PA1-74421) or anti-mouse IgG2a (1:5000, ThermoFisher, #A-10685) for 1 hr at 37 °C. The enzymatic reaction was performed with ELISA TMB stabilized chromogen (Invitrogen) and stopped by ELISA stop solution (Invitrogen). The absorbance at 450 nm was measured by Molecular Devices plate reader SpectraMax iD5. The endpoint titer was calculated as the highest dilution of the serum sample that produced a signal above the cutoff set as 2.1 - fold the value of the blank.

<u>Western Blot</u>

BHK-21 cells were transfected with mRNA vaccines coding for bi- and trivalent HA antigens: 1 µg mRNA /well (6 well plate). After 24 hrs, collected supernatants were separated by SEC-HPLC in PBS mobile phase on Agilent Bio SEC-5 column (1000Å, 7.8 x 300 mm, 5 µm); BEH200 SEC protein standard mix (Waters 186006518-1) was used as a molecular weight standard. The SEC fractions were analyzed by reduced SDS-PAGE and Western blot with influenza A HA rabbit monoclonal antibody (Invitrogen, MA5-30006) and goat anti-rabbit IgG secondary antibody (Invitrogen, 31460).

Supplemental Tables and Figures

Figure	Valency	Construct	Antigen Format	Antigen Source	Amino Acid Positions	Construct Map
3	Movovalent	A_{H1}	Membrane Bound	A/Wisconsin/588/2019	Met1-Ile566	Cap1 – A/Wisconsin22/23 WT HA – Poly A
	Movovalent	A_{H3}	Membrane Bound	A/Darwin/6/2021	Met1-Ile566	Cap1 – A/Darwin WT HA – Poly A
	Movovalent	B_Y	Membrane Bound	B/Phuket/3073/2013	Met1-Leu584	Cap1 – B/Phuket WT HA – Poly A
	Movovalent	B_V	Membrane Bound	B/Austria/1359417/2021	Met1-Leu582	Cap1 – B/Austria WT HA – Poly A
3	Bivalent	B_YB_V	Secreted	B/Phuket/3073/2013	Met1-Thr547	Cap1 – B/Phuket HA ectodomain – B/Austria HA ectodomain – 3'UTR – Poly A
				B/Austria/1359417/2021	Asp16-Ala534	
	Bivalent	$A_{H3}A_{H1}$	Secreted	A/Darwin/6/2021	Met1-Asp529	Cap1 – A/Darwin HA ectodomain – A/Wisconsin22/23 HA ectodomain – 3'UTR – Poly A
				A/Wisconsin/588/2019	Asp18-Asp518	
3,4	Bivalent	$^{(S,L)}B_YA_{H1}$	Secreted	B/Phuket/3073/2013	Met1-Thr547	Cap1 – B/Phuket HA ectodomain – A/Wisconsin23/24 HA ectodomain – 3'UTR – Poly A
				A/Wisconsin/67/2022	Asp18-Asp518	
	Bivalent	$^{(S,L)}B_VA_{H3}$	Secreted	B/Austria/1359417/2021	Met1-Thr545	Cap1 – B/Austria HA ectodomain – A/Darwin HA ectodomain – 3'UTR – Poly A
				A/Darwin/6/2021	Gln17-Gly520	
4	Bivalent	$^{(S,EL)}B_YA_{H1}$	Secreted	B/Phuket/3073/2013	Met1-Thr547	Cap1 – B/Phuket HA ectodomain – A/Wisconsin HA ectodomain – 3'UTR – Poly A
				A/Wisconsin/67/2022	Asp18-Asp518	
	Bivalent	$^{(S,EL)}B_VA_{H3}$	Secreted	B/Austria/1359417/2021	Met1-Thr545	Cap1 – B/Austria HA ectodomain – A/Darwin HA ectodomain – 3'UTR – Poly A
				A/Darwin/6/2021	Gln17-Gly520	
	Bivalent	$^{(M,L)}B_YA_{H1}$	Membrane Bound	B/Phuket/3073/2013	Met1-Thr547	Cap1 – B/Phuket HA ectodomain – A/Wisconsin WT HA – 3'UTR – Poly A
				A/Wisconsin/67/2022	Asp18-Ile566	
	Bivalent	$^{(M,L)}B_VA_{H3}$	Membrane Bound	B/Austria/1359417/2021	Met1-Thr545	Cap1 – B/Austria HA ectodomain – A/Darwin WT HA – 3'UTR – Poly A
				A/Darwin/6/2021	Gln17-Ile566	
	Bivalent	$^{(M,EL)}B_YA_{H1}$	Membrane Bound	B/Phuket/3073/2013	Met1-Thr547	Cap1 – B/Phuket HA ectodomain – A/Wisconsin WT HA – 3'UTR – Poly A
				A/Wisconsin/67/2022	Asp18-Ile566	
	Bivalent	$^{(M,EL)}B_VA_{H3}$	Membrane Bound	B/Austria/1359417/2021	Met1-Thr545	Cap1 – B/Austria HA ectodomain – A/Darwin WT HA – 3'UTR – Poly A
				A/Darwin/6/2021	Gln17-Ile566	
5	Bivalent	$^{(M)}B_VA_{H3}$	Membrane Bound	B/Austria/1359417/2021	Met1-Thr545	Cap1 – B/Austria HA ectodomain – A/Massachusetts WT HA – 3'UTR – Poly A
				A/Massachusetts/18/2022	Gln17-Ile566	
	Bivalent	$^{(M)}A_{H3}A_{H1}$	Membrane Bound	A/Massachusetts/18/2022	Met1-Asp529	Cap1 – A/Massachusetts HA ectodomain – A/Wisconsin WT HA – 3'UTR – Poly A
				A/Wisconsin/67/2022	Asp18-Ile566	
5,6,7	Trivalent	$^{(S)}B_VA_{H3}A_{H1}$	Secreted	B/Austria/1359417/2021	Met1-Thr545	Cap1 – B/Austria HA ectodomain – A/Massachusetts HA ectodomain – A/Wisconsin HA ectodomain – Poly A
				A/Massachusetts/18/2022	Gln17-Asp529	
				A/Wisconsin/67/2022	Asp18-Asp518	
6,7	Bivalent	$^{(S)}COV_{XBB}COV_{XBB}$	Secreted	hCoV-19/USA/RI-CDC-2-6647173/2022	Asn327-Phe537	Cap1 – Omicron XBB.1.5 RBD – Omicron XBB.1.5 RBD – Poly A
				hCoV-19/USA/RI-CDC-2-6647173/2022	Asn327-Phe537	
7	Tetravalent	$COV_{XBB}B_VA_{H3}A_{H1}$	Secreted	hCoV-19/USA/RI-CDC-2-6647173/2022	Asn327-Phe537	Cap1 – Omicron XBB.1.5 RBD – B/Austria HA ectodomain – A/Massachusetts HA ectodomain – A/Wisconsin HA ectodomain – Poly A
				B/Austria/1359417/2021	Asp16-Thr545	
				A/Massachusetts/18/2022	Gln17-Asp529	
				A/Wisconsin/67/2022	Asp18-Asp518	
7	Tetravalent	$B_VA_{H3}A_{H1}COV_{XBB}$	Secreted	B/Austria/1359417/2021	Met1-Thr545	Cap1 – B/Austria HA ectodomain – A/Massachusetts HA ectodomain – A/Wisconsin HA ectodomain – Omicron XBB.1.5 RBD – Poly A
				A/Massachusetts/18/2022	Gln17-Asp529	
				A/Wisconsin/67/2022	Asp18-Asp518	
				hCoV-19/USA/RI-CDC-2-6647173/2022	Asn327-Phe537	

Key	
Identity	Color/Pattern
5' or 3' UTR region	
Homologous signal peptide	
Heterologous signal peptide	
Transmembrane and cytoplasmic domain	
Gly-rich linker	
T4 Foldon domain	

Table S1. Antigen composition.

	Log2 HAI GMT Data for Seasonal Influenza Strains			
Vaccine	B/Victoria	B/Yamagata	H1N1	H3N2
LNP Control	3.32	3.32	3.32	3.32
$A_{H1}+A_{H3}+B_V+B_Y$	9.16	9.16	10.24	10.66
$B_YA_{H1}+B_VA_{H3}$	10.41	9.74	8.24	8.82
$B_YB_V+A_{H3}A_{H1}$	3.82	7.82	9.40	8.99

Note: Post-boost vaccination seroconversion rates reached 100% for all influenza strains across all vaccines, except for B/Victoria in the B_YB_V + $A_{H3}A_{H1}$ mRNA vaccine group in which log 2 HAI GMT of 3.82 corresponded to the seroconversion rate of 33.33%.

Table S2A. Summary of HAI GMT data included in Figure 3.

	Log2 HAI GMT Data for Seasonal Influenza Strains			
Vaccine	B/Victoria	B/Yamagata	H1N1	H3N2
LNP Control	3.32	3.32	3.32	3.32
Fluad	9.57	10.74	10.82	11.91
$^{(S,L)}B_VA_{H3}+B_yA_{H1}$	10.82	10.74	8.91	10.41
$^{(S,EL)}B_VA_{H3}+B_yA_{H1}$	10.91	11.32	10.74	9.66
$^{(M,L)}B_VA_{H3}+B_yA_{H1}$	11.07	10.32	8.91	9.91
$^{(M,EL)}B_VA_{H3}+B_yA_{H1}$	11.32	11.32	10.49	10.24

Note: Post-boost vaccination seroconversion rates reached 100% for all influenza strains across all vaccine groups.

Table S2B. Summary of HAI GMT data included in Figure 4

	Log2 HAI GMT Data for Seasonal Influenza Strains		
Vaccine	B/Victoria	H1N1	H3N2
LNP Control	3.32	3.32	3.32
FluzoneHD	10.01	10.07	11.20
$^{(M)}B_VA_{H3}+A_{H3}A_{H1}$	10.57	12.32	13.38
$^{(S)}B_VA_{H3}A_{H1}$	11.76	12.95	13.20

Note: Post-boost vaccination seroconversion rates reached 100% for all influenza strains across all vaccine groups.

Table S2C. Summary of HAI GMT data included in Figure 5.

	Log2 HAI GMT Data for Seasonal Influenza Strains		
Vaccine	B/Victoria	H1N1	H3N2
LNP Control	3.32	3.32	3.32
Spikevax	3.32	3.32	3.32
FluzoneHD	8.68	8.25	9.25
$B_VA_{H3}A_{H1}$	11.32	11.11	11.32
$B_VA_{H3}A_{H1}$+ $COV_{XBB}COV_{XBB}$ (12 μg)	10.96	11.11	11.82
$B_VA_{H3}A_{H1}$+ $COV_{XBB}COV_{XBB}$ (3 μg)	9.89	9.54	10.25

Note: Post-boost vaccination seroconversion rates reached 100% for all influenza strains across all vaccine groups.

Table S2D. Summary of HAI GMT data included in Figure 6.

	Log2 HAI GMT Data for Seasonal Influenza Strains		
Vaccine	B/Victoria	H1N1	H3N2
LNP Control	3.32	3.32	3.32
Spikevax	3.32	3.32	3.32
$COV_{XBB}COV_{XBB}$	3.32	3.32	3.32
$B_VA_{H3}A_{H1}$+ $COV_{XBB}COV_{XBB}$	11.18	10.18	12.39
$COV_{XBB}B_VA_{H3}A_{H1}$	5.89	9.97	9.82
$B_VA_{H3}A_{H1}COV_{XBB}$	11.18	9.89	12.39

Note: Post-boost vaccination seroconversion rates reached 100% for all influenza strains across all vaccines, except for B/Victoria in the $COV_{XBB}B_VA_{H3}A_{H1}$ mRNA vaccine group in which log 2 HAI GMT of 5.89 corresponded to the seroconversion rate of 57.14%.

Table S2E. Summary of HAI GMT data included in Figure 7.

	RTU LNP	Encapsulated LNP
Instrument	Dolomite Mitos Microfluidics System, Unchained Labs, Pleasanton, CA, USA,	NanoAssemblr® Ignite, Precision NanoSystems Inc. (PNI), Vancouver, BC, Canada
Aqueous solution and lipid mixture solution	Citrate buffer: lipid solution in dehydrated ethanol	Citrate buffer: lipid solution in dehydrated ethanol
Flow Rate Ratio (FRR)	4:1	5:1

Table S3A. Microfluidic mixing for LNP preparation: key process parameters.

	RTU LNP	Encapsulated LNP
Instrument	TFF system, Repligen KrosFlo KR2i	Dialysis and concentration using an ultracentrifugal filter
Molecular Weight Cut-Off (MWCO)	300 kDa	3.5 kDa (dialysis)<br>100 kDa (concentration)
Material	mPES	regenerated cellulose (dialysis)<br>PES (concentration)
Relative Centrifugal Force (RCF)	N/A	2500 g
Transmembrane Pressure (TMP)	1.2 PSI	N/A
Flow Rate	90 mL/min	N/A

Table S3B. Buffer exchange and concentration of LNPs: key process parameters.

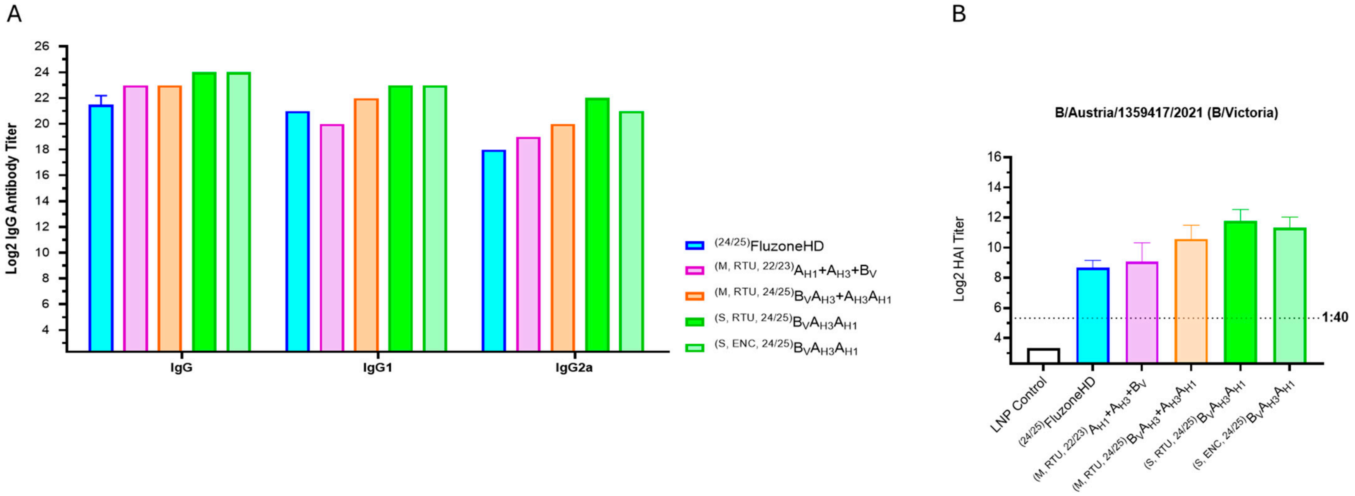

Figure S1: IgG and HAI titers. **(A)** Average total HA-specific IgG, IgG1, and IgG2a titers of pooled D35 mouse sera against B/Austria/1359417/2021 were determined for Fluzone HD (50 µL); RTU formulated$^{(RTU)}$ $A_{H1}+A_{H3}+B_V$ influenza vaccines (2 µg each mRNA) expressing monovalent membrane $^{(M)}$ HA antigens from the 2022/23 season$^{(22/23)}$; RTU formulated $B_VA_{H3}+A_{H3}A_{H1}$ influenza vaccines expressing membrane B/A and A/A HA bivalent dumbbells (5 µg each) from the 2024/25 season$^{(24.25)}$; RTU formulated $B_VA_{H3}A_{H1}$ influenza vaccines (10 µg) expressing secreted$^{(S)}$ trivalent HA dumbbells from the 2024/25 season; Encapsulated$^{(ENC)}$ $B_VA_{H3}A_{H1}$ influenza vaccines (6 µg) expressing secreted trivalent HA dumbbells from the 2024/25 season. **(B)** Day 35 serum HAI GMTs (N =7-8 per group) against B/Austria/1359417/2021 for the same groups as seen in **(A)**.

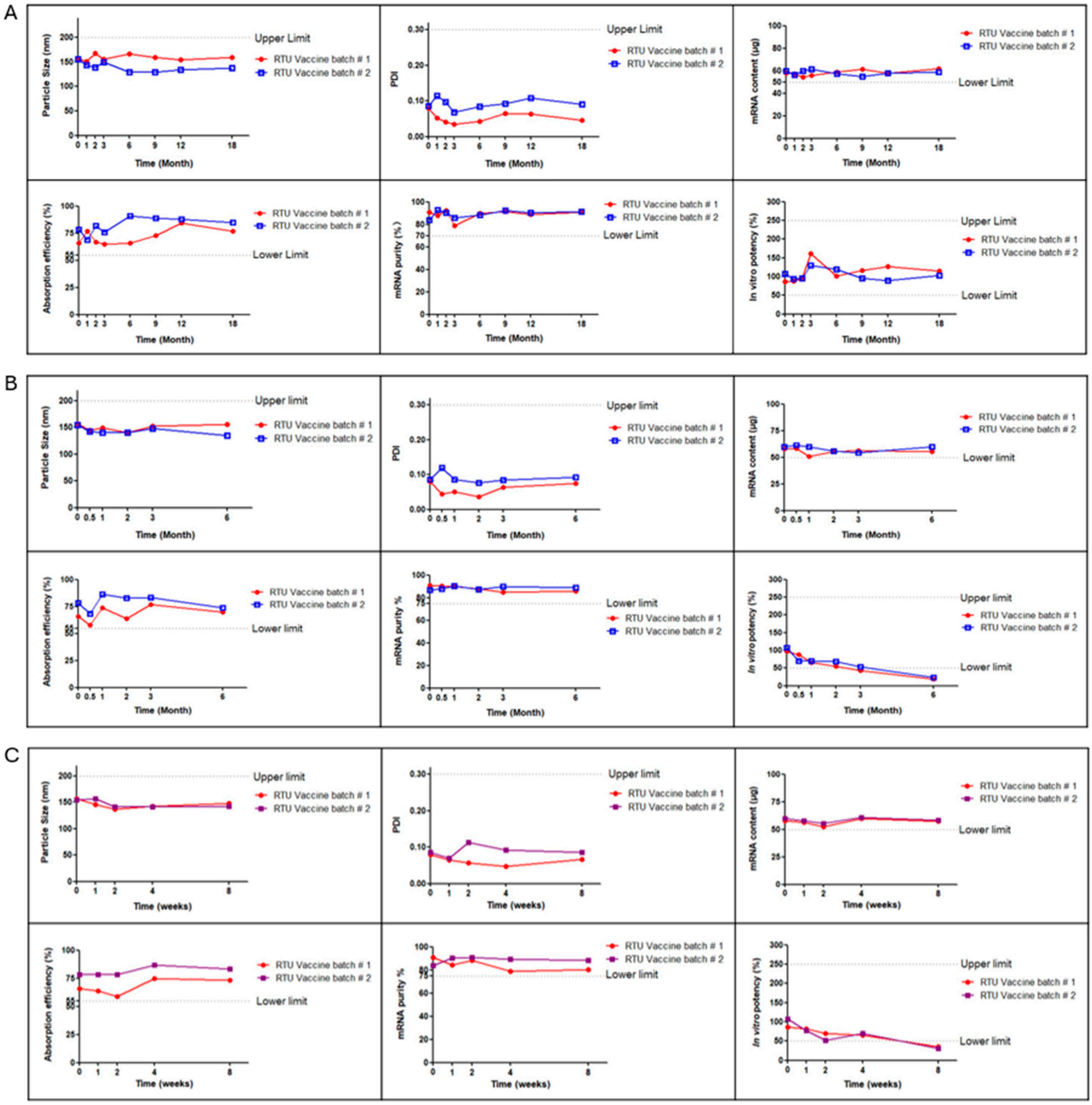

Figure S2: Release and stability testing data for RTU LNP formulation. Stability of lyophylized mRNA component of RTU formulation at **(A)** 2-8 °C, **(B)** 25 °C, and **(C)** 37 °C.

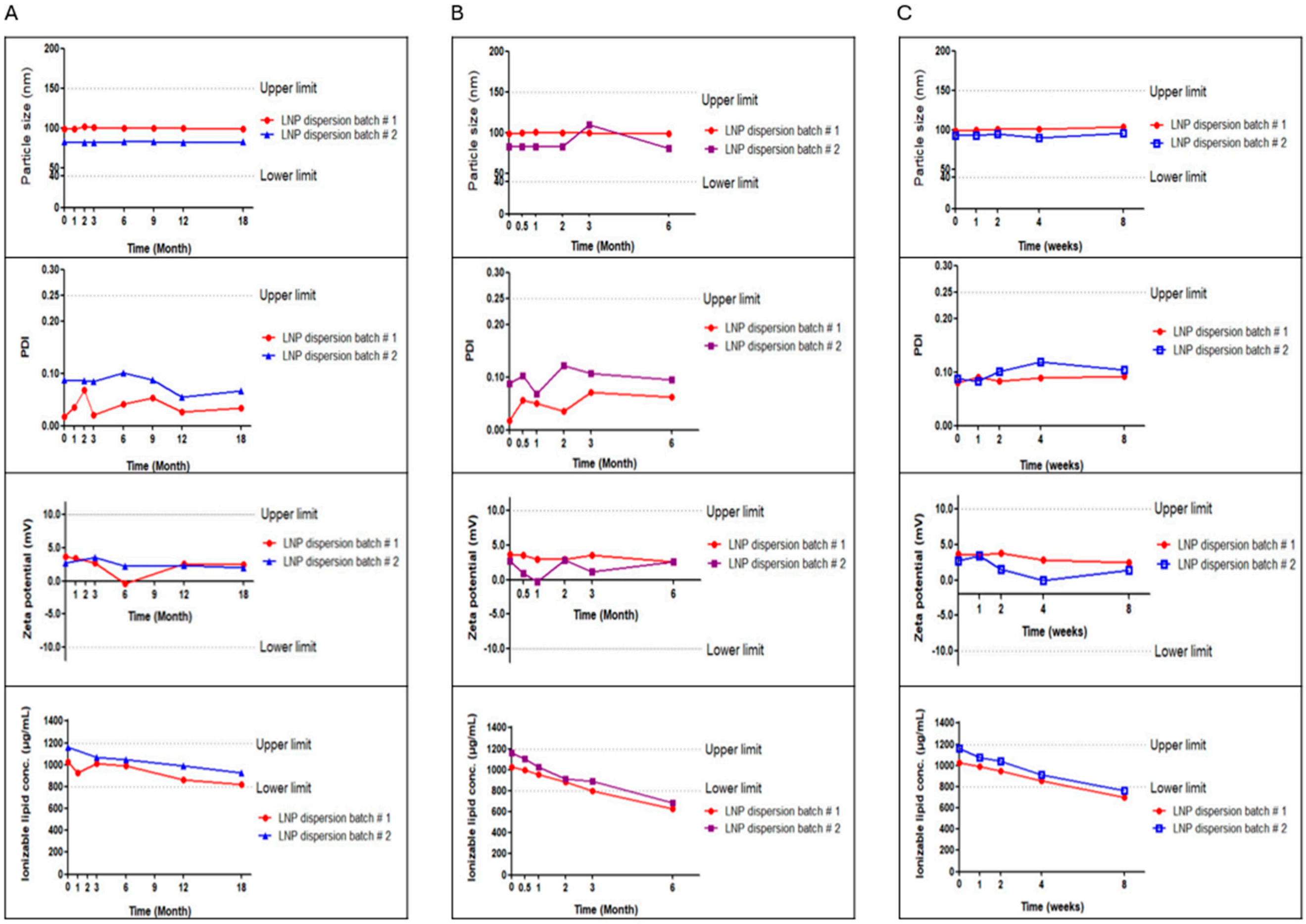

Figure S3: Release and stability testing data for RTU LNP formulation. Stability of LNP dispersion component of RTU formulation at **(A)** 2-8 °C, **(B)** 25 °C, and **(C)** 37 °C.

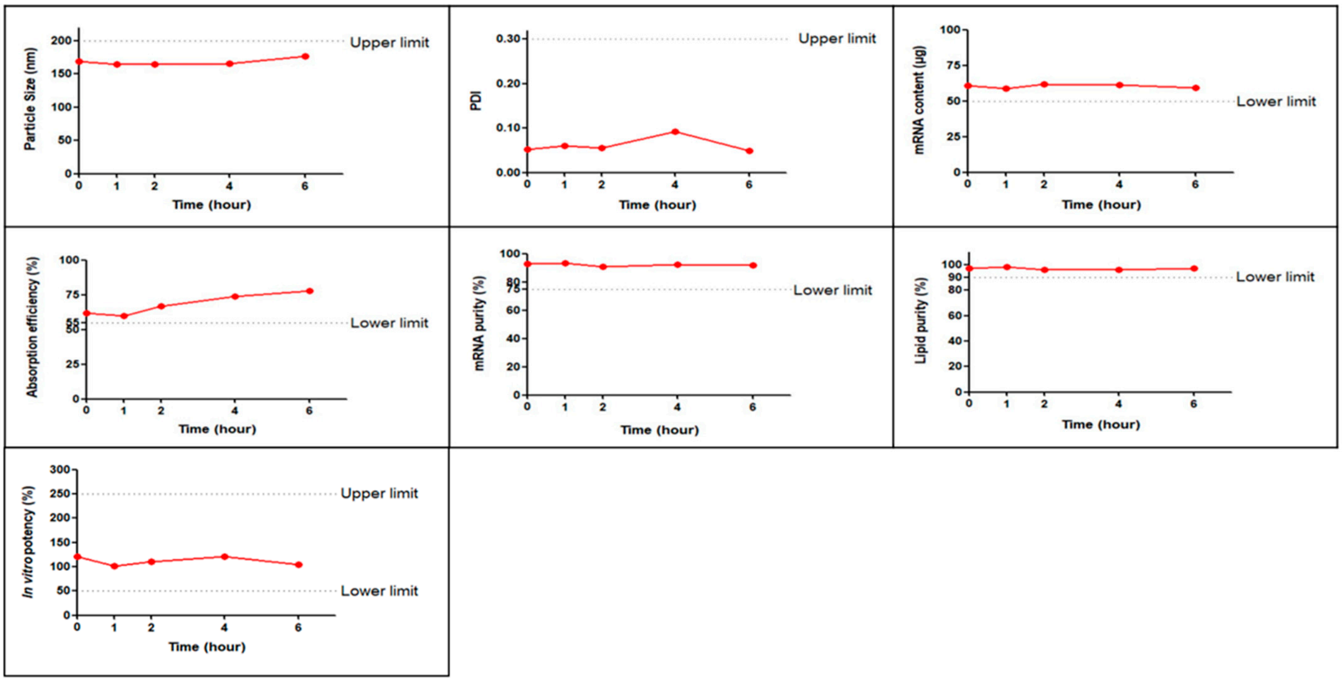

Figure S4: In-use stability of the reconstituted RTU LNP mRNA vaccine at 25±3 °C.

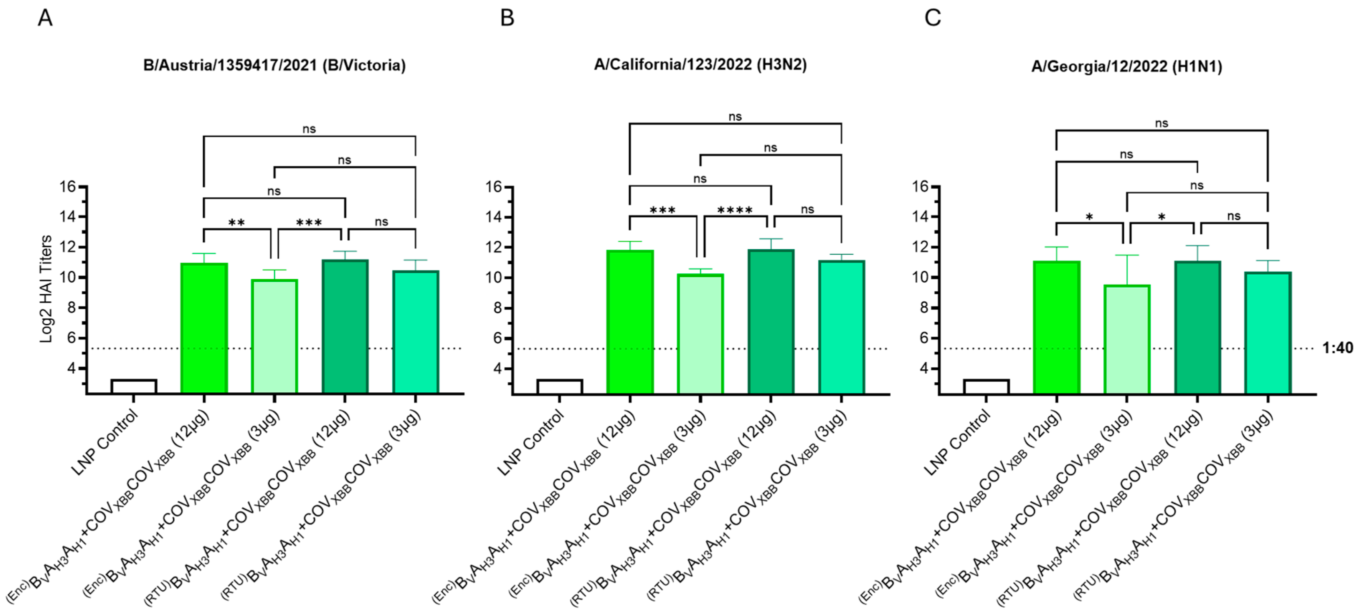

Figure S5: Encapsulated LNP vs. RTU LNP formulation **(A-C)** Day 35 serum HAI GMTs (N = 7 per group) against 2024/25 seasonal influenza viruses were determined for LNP Control (200 µL); Encapsulated(Enc) $B_VA_{H3}A_{H1}$ vaccines tested at low and high doses (3 µg or 12 µg total mRNA) RTU formulated(RTU) $B_VA_{H3}A_{H1}$ vaccines tested at low and high doses (3 µg or 12 µg total mRNA). $P < 0.05$(*), $P < 0.01$(**), $P < 0.001$(***) and $P < 0.0001$(****) indicate statistically significant differences; not significant (n.s.)

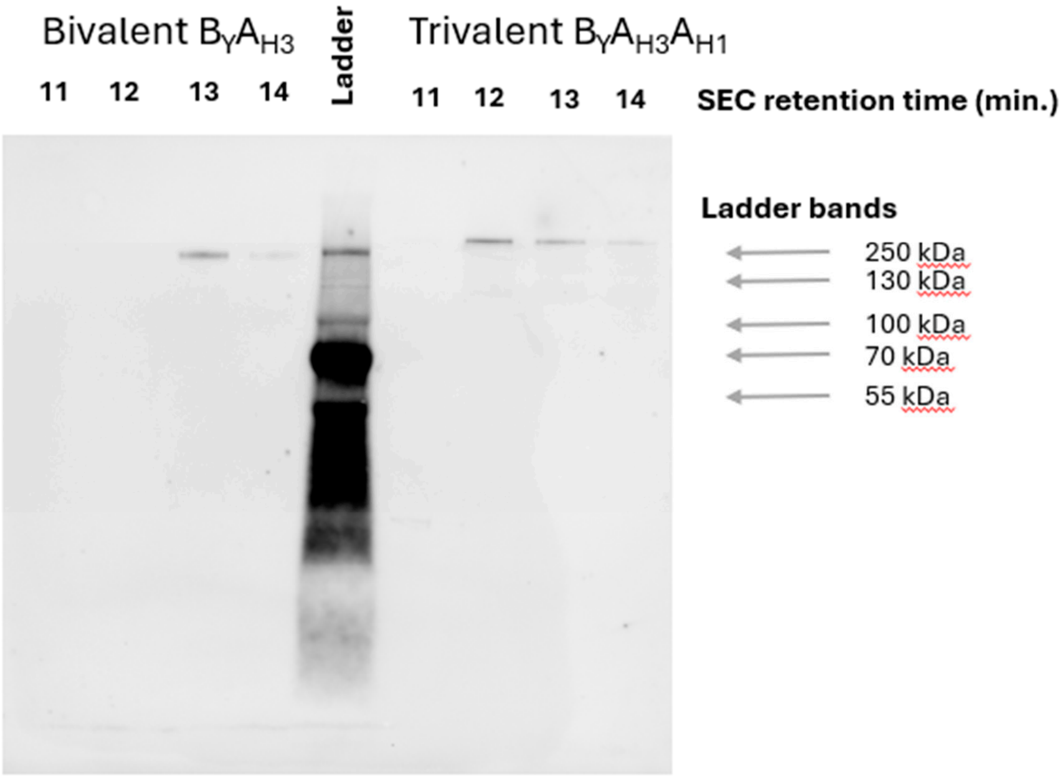

Figure S6. Western blot analysis of bivalent and trivalent HA dumbbell antigens. Each lane represents the SEC retention time (min.) for either a bivalent or trivalent HA dumbbell. The differences in the theoretical molecular weights of non-glycosylated bi- and trivalent HA antigens, 120 and 183 kDa, respectively, were reflected by the shift in SDS-PAGE migration of the HA bands. The HA SEC peaks in 13-14 min. fraction (for B_YA_{H3}) and 12-13 min. fraction (for $B_YA_{H3}A_{H1}$) had the estimated molecular weights in 150-660 kDa range and above 660 kDa, respectively.

Supplemental Information

Nucleotide Sequence of $B_VA_{H3}A_{H1}$ mRNA

Legend:
5' UTR (mutant variant of the chicken beta-globin gene UTR; GenBank: V00409.1), **native signal peptide of HA from B/Austria/1359417/2021**, B/Austria/1359417/2021 HA ectodomain, linker, T4 foldon, linker, A/Massachusetts/18/2022 HA ectodomain, linker, T4 foldon, linker, A/Wisconsin/67/2022 HA ectodomain, *3' UTR*, hybrid poly(A)

GGACAAUUACAAUACAAGCGAGCUAGACAAGCUCGCUAGACCGCCACCGCCACC**AUGAAGGCCAUCAUCGUGCUGCUGAUGGUG**
GUGACAAGCAACGCCGACAGAAUCUGCACCGGCAUCACAAGCAGCAACAGCCCCCACGUGGUGAAAACCGCGACACAAGGCGAG
GUGAACGUGACCGGCGUGAUCCCCCUGACCACCACCCCCACCAAGAGCCACUUCGCCAACCUGAAGGGCACCGAGACAAGAGGC
AAGCUGUGCCCCAAGUGCCUGAACUGCACCGACCUGGACGUGGCCCUGGGCAGACCCAAGUGCACCGGCAAGAUCCCUAGCGCU
AGAGUGAGCAUCCUGCACGAGGUGAGACCCGUGACAAGCGGCUGCUUCCCCAUCAUGCACGACAGAACCAAGAUCAGACAGCUG
CCCAACCUGCUGAGAGGCUACGAGCACGUGAGACUGAGCACCCACAACGUGAUCAACACCGAGGACGCCCCCGGCGGCCCCUAC
GAGAUCGGCACAAGCGGCAGCUGUCUGAAUAUCACCAACGGAAAGGGCUUCUUCGCCACCAUGGCCUGGGCCGUGCCCAAGAAC
AAGACCGCUACGAACCCCCUGACCAUCGAGGUGCCCUACAUCUGCACCGAGGAGGAGGAUCAGAUCACCGUGUGGGGCUUCCAC
AGCGACGACGAGACACAGAUGGCUAGACUGUACGGCGACAGCAAGCCUCAGAAGUUCACAAGCAGCGCCAACGGCGUGACCACC
CACUACGUGUCUCAGAUCGGCGGCUUCCCCAAUCAGACCGAGGACGGCGGCCUGCCUCAGAGCGGCAGAAUCGUGGUGGACUAC

AUGGUGCAAAAGAGCGGCAAGACCGGCACCAUCACCUAUCAGAGAGGCAUCCUGCUGCCUCAGAAGGUGUGGUGCGCUAGCGGC
AAGAGCAAGGUGAUCAAGGGCAGCCUGCCCCUGAUCGGCGAGGCCGACUGCCUGCACGAGAAGUACGGCGGCCUGAACAAGAGC
AAGCCCUACUACACCGGCGAGCACGCCAAGGCCAUCGGCAACUGCCCCAUCUGGGUGAAGACCCCCCUGAAGCUGGCCAACGGC
ACCAAGUACAGACCCCCCGCCAAGCUGCUGAAGGAGAGAGGAUUCUUUGGAGCCAUCGCCGGCUUCCUGGAGGGAGGCUGGGAA
GGUAUGAUAGCCGGGUGGCACGGCUAUACUAGCCACGGCGCUCAUGGCGUGGCCGUGGCCGCCGACCUCAAGAGCACCCAAGAG
GCCAUCAACAAGAUCACCAAGAACCUGAACAGCCUGAGCGAGCUGGAGGUGAAGAACCUGCAGAGACUGAGCGGCGCCAUGGAC
GAGCUGCACAACGAGAUCCUGGAGCUGGACGAGAAGGUGGACGACCUGAGAGCCGACACCAUCAGCUCUCAGAUCGAGCUGGCC
GUGCUGCUGAGCAACGAGGGCAUCAUCAACAGCGAGGACGAGCACCUGCUGGCCCUGGAGAGAAAGCUGAAGAAGAUGCUGGGC
CCUAGCGCCGUGGAGAUCGGCAACGGCUGCUUCGAGACCAAGCACAAGUGCAAUCAGACCUGCCUGGACAGAAUCGCCGCCGGC
ACCUUCGACGCCGGCGAGUUCAGCCUGCCCACCUUCGACAGCCUGAACAUCACCGCCGCGAGCCUGAACGACGACGGCCUGGAC
AACCACACCGGAGGAAGCGGAGGAGGAAGCGGCUACAUCCCCGAGGCCCCUAGAGACGGCCAAGCCUACGUGAGAAAGGACGGC
GAGUGGGUGCUGCUGAGCACCUUCCUGGGAUCCGGUGGAGGAGGGUCAGGCGGUGGAGGCUCUGGUGGAGGUGGAAGUGGAGGA
GGUGGUAGUGGUGGUGGAGGUUCCGGAGGUGGUGGCAGUGGCGGCGGGGGAUCACAGAAAAUCCCAGGCAAUGAUAACAGCACG
GCCACCCUGUGCCUGGGCCACCACGCCGUGCCCAACGGCACCAUCGUGAAAACCAUCACCAACGAUAGAAUCGAGGUGACCAAC
GCUACAGAGCUGGUGCAGAACAGUUCUAUCGGCAAGAUUUGUAAUAGUCCUCAUCAGAUCCUGGACGGAGGCAACUGCACCCUG
AUCGAUGCUCUGCUGGGCGAUCCUCAGUGUGACGGCUUUCAAAACAAAGAGUGGGACCUUUUCGUGGAAAGAAGCAGAGCCAAC
AGCUCCUGCUACCCCUACGACGUGCCUGACUACGCCAGCCUGAGGAGCCUGGUGGCUUCUAGCGGCACCCUGGAAUUCAAAAAC
GAGAGCUUUAACUGGACCGGCGUGAAACAGAACGGCACCAGCUCUGCCUGCAAGCGGGGAAGCAGCAGCAGCUUCUUCAGCCGG
CUGAAUUGGCUGACCUCCCUGAACAACAUCUAUCCUGCCCAGAACGUGACCAUGCCUAACAAGGAACAGUUCGACAAGCUGUAC
AUCUGGGGCGUGCACCACCCCGAUACCGAUAAGAACCAGUUCUCCCUGUUUGCCCAGAGCAGCGGCAGAAUCACAGUGUCCACC
AAGAGAAGCCAGCAGGCUGUUAUUCCCAACAUCGGCAGCAGACCUAGAGUGCGGGACAUCCCAUCUAGAAUCAGCAUCUACUGG
ACAAUCGUGAAGCCCGGCGACAUCCUGCUGAUCAACUCUACCGGCAAUCUGAUCGCCCCUAGAGGCUACUUCAAAAUCCGGUCU
GGCAAGUCCUCUAUCAUGCGGAGCGACGCCCCUAUCGGCAAGUGCAAGAGCGAGUGCAUCACCCCUAACGGCUCUAUCCCCAAU
GACAAGCCAUUCCAGAACGUGAACCGGAUUACAUACGGCGCCUGUCCUAGGUACGUGAAGCAAAGCACACUGAAACUGGCCACA
GGCAUGAGAAACGUGCCCGAAAAGCAGACCAGAGGCAUCUUCGGCGCCAUCGCCGGAUUCAUCGAGAACGGAUGGGAGGGCAUG
GUCGACGGAUGGUACGGCUUCCGGCACCAGAAUUCUGAAGGCAGAGGCCAGGCCGCCGACCUGAAGUCUACCCAAGCAGCUAUC
GACCAGAUUUCUGGCAAGUUGAACCGCCUGAUCGGUAAGACAAACGAGAAGUUCCACCAAAUCGAGAAGGAAUUCAGCGAGGUG
GAAGGAAGAGUCCAGGACCUUGAAAAGUACGUGGAAGAUACAAAGAUCGAUCUGUGGUCCUACAACGCCGAGCUGCUGGUCGCC
CUGGAAAAUCAGCACACCAUCGACCUGACCGACAGCGAGAUGAACAAGCUGUUCGAGAAAACAAAAAAGCAGCUGCGGGAAAAC
GCCGAGGACAUGGGCAAUGGUUGUUUUAAGAUCUACCACAAGUGCGACAACGCUUGCAUCGGCAGCAUCAGAAACGAAACAUAC
GACCACAACGUGUACCGGGACGAGGCCCUGAACAACAGGUUCCAGAUCAAGGGCGUGGAACUGAAGUCUGGCUACAAGGACGGC
GGCAGCGGCGGGGGCAGCGGCUACAUCCCCGAGGCCCCUAGAGACGGCCAAGCCUACGUGAGAAAGGACGGCGAGUGGGUGCUG
CUGAGCACCUUCCUGGGAUCCGGUGGAGGAGGGUCAGGCGGUGGAGGCUCUGGUGGAGGUGGAAGUGGAGGAGGUGGUAGUGGU
GGUGGAGGUUCCGGAGGUGGUGGCAGUGGCGGCGGGGGAUCAGACACCCUGUGUAUCGGAUAUCAUGCCAACAACAGCACAGAU
ACAGUGGACACCGUGCUGGAAAAGAACGUCACCGUGACCCACAGCGUCAACCUCCUGGAGGACAAGCACAACGGCAAGCUCUGC
AAGCUCCGGGGAGUGGCCCCUCUGCACCUGGGCCAGUGUAAUAUCGCCGGCUGGAUCCUGGGAAAUCCUGAGUGUGAGUCUCUG
UCCACCGCCAGAAGCUGGUCCUACAUUGUGGAGACAAGCAACAGCGACAACGGCACCUGCUACCCUGGCGACUUCAUCAACUAC
GAGGAACUCCGCGAGCAGCUGUCUUCGGUGUCUAGCUUUGAGCGGUUCGAAAUCUUCCCCAAGACCUCUAGCUGGCCUAACCAC
GACAGCGAUAAUGGCGUUACAGCCGCCUGCAGCCACGCCGGCGCGAGGUCCUUCUACAAGAACCUGAUCUGGCUGGUGAAGAAG
GGGAAGUCUUAUCCUAAGAUUAACCAGACCUACAUCAAUGACAAAGGCAAAGAAGUGCUGGUGCUGUGGGGCAUCCACCACCCC
CCCACCAUCACCGACCAGGAGAGCCUGUAUCAGAACGCCGACGCCUACGUGUUCGUGGGCACCAGCCGGUACAGCAAGAAAUUC
AAGCCUGAAAUCGCCACAAGACCUAAAGUGCGGGACCAGGCCGGAAGAAUGAACUACUACUGGACCCUGGUUGAGCCUGGAGAU
AAGAUCACAUUCGAGGCUACAGGCAACCUGGUGGCUCCAAGAUACGCCUUCACUAUGGAAAAAGAGGCCGGAAGCGGCAUCAUC
AUCAGCGAUACCCCAGUGCACGACUGCAACGCUACAUGCCAGACCCCCGAGGGCGCCAUUAACACCAGCUUGCCUUUCCAGAAC
GUGCACCCUAUCACAAUUGGCAAGUGUCCUAAGUACGUGAGAUCUACCAAGCUGAGACUGGCCACAGGCCUGCGGAACGUGCCC
AGCAUCCAGUCUAGAGGCCUGUUCGGCGCCAUCGCCGGCUUCAUCGAGGGCGGCUGGACAGGCAUGGUGGAUGGCUGGUACGGC
UACCAUCACCAGAACGACCAAGGCAGCGGCUACGCCGCUGAUCUGAAGAGUACCCAGAAUGCUAUCGAUAAGAUCACAAACAAG
GUGAACAGCGUGAUCGAGAAAAUGAACACACAAUUUACCGCCGUGGGAAAGGAAUUCAACCACCUGGAAAAAGAAUCGAGAAC
CUGAACAAGAAGGUGGACGACGGCUUUCUGGACGUGUGGACCUACAACGCAGAGCUGCUGGUCCUGCUGGAAAACGAGAGAACC
CUGGAUUACCACGAUUCCAACGUGAAAAAUCUGUACGAGAAGGUGCGGCACCAGCUGAAGAACAACGCCAAGGAGAUCGGCAAU
GGUUGUUUUGAGUUCUACCACAAGUGCGACAAUACCUGCAUGGAAUCUGUGAAGAACGGAACCUACGACUACCCCAAGUACAGC
GAGGAAGCCAAGCUGAAUAGAGAAAAGAUCGACUGAUAAGCGGCCGCGCACCAGCACCAAAGAUCACGGAGCACCUACAACCAU
UGCAUGCACCUGCAGAAAUGCUCCGGAGCUGACAGCUUGUGACAAAUAAAGUUCAUUCAGUGACACUCAACCGGUAAAAAAAAA
AAAAAAAAAAAAAAAAAAAAAUUAAAAAAAAAAAAAAAAAAAAAAAAAAAAAAAAAUUAAAAAAAAAAAAAAAAAAAAAAAAAAAAA
AUUAAAAAAAAAAAAAAAAAAAAAAAAAAAAAA

Amino Acid Sequence of $B_V A_{H3} A_{H1}$ Antigen

MKAIIVLLMVVTSNADRICTGITSSNSPHVVKTATQGEVNVTGVIPLTTTPTKSHFANLKGTETRGKLCPKCLNCTDLDVALGR
PKCTGKIPSARVSILHEVRPVTSGCFPIMHDRTKIRQLPNLLRGYEHVRLSTHNVINTEDAPGGPYEIGTSGSCLNITNGKGFF
ATMAWAVPKNKTATNPLTIEVPYICTEEEDQITVWGFHSDDETQMARLYGDSKPQKFTSSANGVTTHYVSQIGGFPNQTEDGGL

PQSGRIVVDYMVQKSGKTGTITYQRGILLPQKVWCASGKSKVIKGSLPLIGEADCLHEKYGGLNKSKPYYTGEHAKAIGNCPIW
VKTPLKLANGTKYRPPAKLLKERGFFGAIAGFLEGGWEGMIAGWHGYTSHGAHGVAVAADLKSTQEAINKITKNLNSLSELEVK
NLQRLSGAMDELHNEILELDEKVDDLRADTISSQIELAVLLSNEGIINSEDEHLLALERKLKKMLGPSAVEIGNGCFETKHKCN
QTCLDRIAAGTFDAGEFSLPTFDSLNITAASLNDDGLDNHTGGSGGGSGYIPEAPRDGQAYVRKDGEWVLLSTFLGSGGGGSGG
GGSGGGGSGGGGSGGGGSGGGGSGGGGSQKIPGNDNSTATLCLGHHAVPNGTIVKTITNDRIEVTNATELVQNSSIGKICNSPH
QILDGGNCTLIDALLGDPQCDGFQNKEWDLFVERSRANSSCYPYDVPDYASLRSLVASSGTLEFKNESFNWTGVKQNGTSSACK
RGSSSSFFSRLNWLTSLNNIYPAQNVTMPNKEQFDKLYIWGVHHPDTDKNQFSLFAQSSGRITVSTKRSQQAVIPNIGSRPRVR
DIPSRISIYWTIVKPGDILLINSTGNLIAPRGYFKIRSGKSSIMRSDAPIGKCKSECITPNGSIPNDKPFQNVNRITYGACPRY
VKQSTLKLATGMRNVPEKQTRGIFGAIAGFIENGWEGMVDGWYGFRHQNSEGRGQAADLKSTQAAIDQISGKLNRLIGKTNEKF
HQIEKEFSEVEGRVQDLEKYVEDTKIDLWSYNAELLVALENQHTIDLTDSEMNKLFEKTKKQLRENAEDMGNGCFKIYHKCDNA
CIGSIRNETYDHNVYRDEALNNRFQIKGVELKSGYKDGGSGGGSGYIPEAPRDGQAYVRKDGEWVLLSTFLGSGGGGSGGGGSG
GGGSGGGGSGGGGSGGGGSGGGGSDTLCIGYHANNSTDTVDTVLEKNVTVTHSVNLLEDKHNGKLCKLRGVAPLHLGQCNIAGW
ILGNPECESLSTARSWSYIVETSNSDNGTCYPGDFINYEELREQLSSVSSFERFEIFPKTSSWPNHDSDNGVTAACSHAGARSF
YKNLIWLVKKGKSYPKINQTYINDKGKEVLVLWGIHHPPTITDQESLYQNADAYVFVGTSRYSKKFKPEIATRPKVRDQAGRMN
YYWTLVEPGDKITFEATGNLVAPRYAFTMEKEAGSGIIISDTPVHDCNATCQTPEGAINTSLPFQNVHPITIGKCPKYVRSTKL
RLATGLRNVPSIQSRGLFGAIAGFIEGGWTGMVDGWYGYHHQNDQGSGYAADLKSTQNAIDKITNKVNSVIEKMNTQFTAVGKE
FNHLEKRIENLNKKVDDGFLDVWTYNAELLVLLENERTLDYHDSNVKNLYEKVRHQLKNNAKEIGNGCFEFYHKCDNTCMESVK
NGTYDYPKYSEEAKLNREKID*
